# Phospholipid prodrug conjugates of insoluble chemotherapeutic agents for ultrasound targeted drug delivery

**DOI:** 10.7150/ntno.37738

**Published:** 2020-01-01

**Authors:** Mendi G. Márquez, Rachel Dotson, Sally Pias, Liliya V. Frolova, Michaelann S. Tartis

**Affiliations:** 1 Departments of Chemistry, New Mexico Institute of Mining and Technology, 801 Leroy Place, Socorro, NM 87801, USA; 2 Materials Engineering, New Mexico Institute of Mining and Technology, 801 Leroy Place, Socorro, NM 87801, USA; 3 Chemical Engineering, New Mexico Institute of Mining and Technology, 801 Leroy Place, Socorro, NM 87801, USA

**Keywords:** prodrug-loaded liposomes, podophyllotoxin, microbubbles, ultrasound, lipid, targeted drug delivery

## Abstract

The hydrophobicity and high potency of many therapeutic agents makes them difficult to use effectively in clinical practice. This work focuses on conjugating phospholipid tails (2T) onto podophyllotoxin (P) and its analogue (N) using a linker and characterizing the effects of their incorporation into lipid-based drug delivery vehicles for triggered ultrasound delivery. Differential Scanning Calorimetry results show that successfully synthesized lipophilic prodrugs, 2T-P (~28 % yield) and 2T-N(~26 % yield), incorporate within the lipid membranes of liposomes. As a result of this, increased stability and incorporation are observed in 2T-P and 2T-N in comparison to the parent compounds P and N. Molecular dynamic simulation results support that prodrugs remain within the lipid membrane over a relevant range of concentrations. 2T-N's (IC_50_: 20 nM) biological activity was retained in HeLa cells (cervical cancer), whereas 2T-P's (IC_50_: ~4 µM) suffered, presumably due to steric hindrance. Proof-of-concept studies using ultrasound *in vitro* microbubble and nanodroplet delivery vehicles establish that these prodrugs are capable of localized drug delivery. This study provides useful information about the synthesis of double tail analogues of insoluble chemotherapeutic agents to facilitate incorporation into drug delivery vehicles. The phospholipid attachment strategy presented here could be applied to other well suited drugs such as gemcitabine, commonly known for its treatment of pancreatic cancer.

## Introduction

Delivering chemotherapy to irresectable tumors, such as pancreatic ductal adenocarcinoma or glioblastoma, has so far proved difficult due to physical barriers preventing systemically administered drugs from reaching target tissue 1, 2. It may be possible to efficiently supply an effective dose to target tissues with ultrasound, while minimizing the total administered dose, by utilizing a combination of ultrasound contrast agents and therapeutic agents. By incorporating a chemotherapeutic drug directly into ultrasonically activatable and detectable particles, there is potential for simultaneous monitoring and treatment of vascular tumors with one systemically injected agent. Currently, there are significant challenges associated with limited drug loading and premature release of therapeutics carried within lipid-based carriers, such as liposomes, microbubbles, and nanodroplets 3-8. Drug anchoring strategies aim to overcome this challenge by conjugating highly toxic, hydrophobic compounds to lipophilic moieties for insertion into the lipid-based carriers' membranes 9-11. Building upon this general anchoring concept, our lipid prodrug strategy could be applied to lipid-based carriers to increase drug payload, prevent drug leakage, and employ ultrasound technologies to trigger local delivery.

Gas-filled lipid microbubbles are used as ultrasound contrast agents for imaging the vascularity and perfusion in tumors to monitor treatment 4, 12. However, these agents are not therapeutic. Loading therapeutic compounds into microbubbles has been investigated as a method of particulate drug and gene delivery (for example using targeting ligands or chemical or biological triggers specific to tumor environments 10, 13) and takes advantage of the non-invasive spatial targeting that ultrasound provides. Advances in focused ultrasound, such as ablative high intensity focused ultrasound (HIFU), highlight the non-invasive and extracorporeal nature of therapeutic ultrasound with and without microbubbles 14-16. At lower intensities, ultrasound can induce sonoporation, where cell membrane permeability is temporarily and locally increased, allowing otherwise impermeable agents to enter cells 3, 17-20. A recent clinical study using ultrasound sonoporation demonstrates the feasibility and safety of microbubble-based approaches 11. In order to take advantage of these strategies, it is necessary to tether a large number of potent molecules to a lipid particle in a way that prevents premature release, yet does not prevent the drug from having a therapeutic effect.

Liposomes cannot typically be visualized using ultrasound and are primarily utilized as drug delivery vehicles. Liposomes can encapsulate various types of drugs (both hydrophilic and hydrophobic) within an aqueous core or within the outer membrane. Liposome-based drug delivery vehicles can deliver chemotherapeutic drugs, such as Irinotecan (treats pancreatic cancer, overall 5-year survival of <3%) 21, 22. Unfortunately, the encapsulated drugs have been shown to affect the membrane packing and release kinetics of the liposomes, which results in loading limitations and leaky molecules. In studies of Doxil® (doxorubicin-loaded liposomes used for chemotherapeutic applications), insufficient Doxorubicin-entrapment in the bilayer resulted in early release, hindering the targeted delivery objective 23, 24. Drug-loaded liposomes could be attached to microbubbles and targeted drug release could be accomplished by ultrasound-induced sonoporation; unfortunately, in early models, loading limitations were still present and extensive preparation protocols deemed these formulations clinically unfavorable 25-30. In another approach to improving lipid delivery vehicles, compounds such as Mitomycin-C have been modified with a single PEGylated chain designed to intercalate in the lipid layer as a lipid component. Similarly, cytarabine's conjugation to a lauric acid moiety was shown to increase bioavailability by increasing liposomal stability in the presence of plasma 10, 11, 31.

This approach - synthesizing prodrugs that can effectively incorporate into lipid membranes - shows promise as the next step in the design of more effective liposomal drug-delivery agents. Prodrug anchoring allows a drug molecule to take advantage of existing particulate drug strategies: the potent parent compound is released after intracellular uptake and enzymatic cleavage. Mitomycin-C and doxorubicin (docosahexaenoic acid-DOX conjugates) have each successfully been synthesized into lipid drug conjugates via dithiobenzyl or hydrazine linkers 26, 32, 33. These 'single-tailed' lipophilic lipid drug conjugates have enhanced pharmacokinetics and improved solubility but display low incorporation within lipid-based carriers. Phospholipid-linked or 'two-tailed' prodrugs are generally coupled at the phosphate group or glycerol backbone, aiding in overcoming transport resistant barriers and reducing efflux 26. In this study, we test a phospholipid anchoring strategy that utilizes a linker to tether the drug to the phospholipid, allowing more options for the drug to be spatially located and oriented, while remaining covalently associated with a lipid vehicle. This approach minimizes packing defects, increases incorporation efficiency, and reduces the requirement for purification steps after self-assembly, and can be applied to create a broad range of lipid-based particle types: microbubbles, liposomes, and nanodroplets.

**Figure 1:**
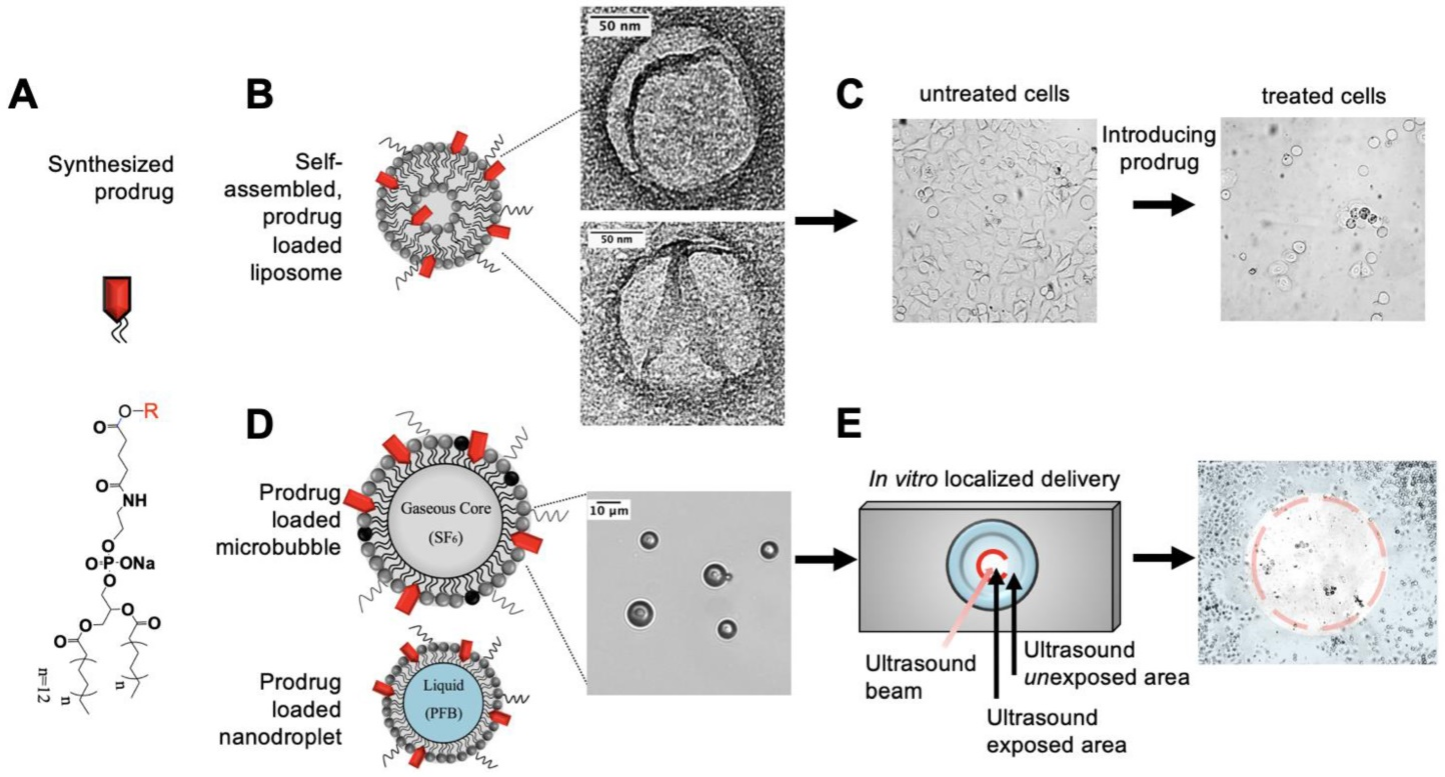
Overview of experimental pathway for synthesized prodrug to *in vitro* localized delivery.** (A)** Anticancer prodrugs were synthesized for incorporation into lipid delivery vehicles. **(B,C)** Characterization studies were performed primarily using liposomes then completed with **(D,E)** microbubbles and nanodroplets for targeted drug delivery with ultrasound. Transmission electron and light microscopy images verify the size and morphology of 20 mol% 2T-N loaded (B) liposomes and (D) microbubbles.

We synthesized lipid prodrugs (2T-P and 2T-N) of podophyllotoxin (P) and its analogue 7-(3,5-Dibromophenyl)-2-hydroxy-7,11-dihydrobenzo[h]-furo[3,4-b]quinolin-8(10H)-one (N) for incorporation into lipid carriers, Figure 1. P and N were chosen due to their hydrophobicity and potent anticancer properties in addition to both inhibit tubulin polymerization, which has been shown to induce apoptosis by inhibiting the microtubule dynamics required for cell division and DNA segregation 34. By anchoring drugs with functionalized phospholipids to a particle, we explore the ability to overcome loading, retention, and purification challenges 35, 36. The lipid prodrugs 2T-P and 2T-N self-assemble into lipid-based particles and require relatively little purification. These drugs can then take advantage of targeting strategies developed for various lipid drug delivery vehicles including active, passive, stealth, and ultrasound triggered release 37. We incorporated these prodrugs into liposomes, microbubbles, and nanodroplets to experimentally investigate their efficacy; to complement our experimental results we carried out atomistic molecular dynamics simulations of lipid bilayers incorporating the prodrugs.

## Materials and Methods

### Chemicals and Materials

3- aminopyrazole, 4- dimethylaminopyridine (DMAP), 5- bromovanillin, dimethyl sulfoxide (DMSO), dimethylformamide (DMF), ethanol (EtOH), methanol (MeOH), methylene chloride (CH_2_Cl_2_), MTT reagent, phosphate buffered saline (PBS), podophyllotoxin (P), tetronic acid, and triethylamine (Et3N) were purchased from Sigma-Aldrich or Fisher Scientific (Milwaukee, WI/Fairlawn,NJ). Chloroform solutions of 1,2-dipalmitoyl-sn-glycero-3-phophate (monosodium salt) (DPPA); 1,2-dipalmitoly-snglycero-3-phosphocholine (DPPC); 1,2-distearoyl-sn-glycerol-3-phosphoethanolamin-N-[methoxy(polyethylene glycol) -2000] ammonium salt (DSPE-PEG2000); and 1,2-distearoyl-sn-glycero-3-phospho-ethanolamine-N-(polyethyleneglycol)-5000) (ammonium salt) (DSPE-PEG5000) were purchased from Avanti Polar Lipids (Alabaster, AL). COATSOME® FE-6060GL (DPPE-Glu) was purchased from NOF America Corporation.

### Synthetic methods for parent compounds and prodrugs

N was synthesized following the procedure presented in Magedov 2011^34^. The parent compound (0.24 mmol, 1 eq.), DCC (0.73 mmol, 3 eq.), DPPE-Glu (0.24 mmol, 1 eq.) and DMAP (0.048 mmol, 0.4 eq.) were combined in a 10 mL flask. 5.5 mL of dry THF was added under nitrogen. The coupling reaction ran at room temperature for 24 hours. Thin layer chromatography (TLC) (precoated silica gel 60F254 glass-backed plates, 250 mm) was used to monitor the reactions and guide all flash column chromatography (Kiesel gel 60, 230-400 mesh). ^1^H and ^13^C NMR were recorded on Jeol Eclipse 300 or Bruker Avance III 400 spectrometers. HRMS analyses were performed at the mass spectrometry facilities of the University of New Mexico and Montana University. Samples were run on an LCT Premier TOF mass spectrometer.

### Liposome preparation

Control and prodrug-loaded lipid films were prepared with chloroform solutions of 1,2-dipalmitoyl- sn- glycero-3-phosphocholine (DPPC) and 1,2-distearoyl -sn- glycero -3- phosphoethanolamine -N- (methoxy (polyethyleneglycol) 2000) ammonium salt (DSPE-PEG2000) mixed with the prodrug solution in chloroform at the desired lipid ratio [DPPC: DSPE-PEG2000: prodrug or drug]. The lipid mixture was then dried under nitrogen gas and further under vacuum at 50 ◦C for 2 h. The prodrug enriched lipid films were resuspended in 1 mL aliquots of 1X phosphate buffer saline (PBS) solution via sonication bath for 30 min at 50 ◦C, resulting in a 1 mg/mL liposome suspension.

### Differential scanning calorimetry

Prodrug-loaded liposome samples were prepared at 20 mg/mL in deionized water for each compound with increasing prodrug concentrations without extrusion. Deionized water was used as the calibration standard. 10 µL from each liposome suspension were transferred and sealed in an aluminum DSC pan then measurements began at room temperature then heated from 15 °C to 55 °C at 5 °C/min. All liposome suspensions used for DSC analysis were prepared in deionized water, instead of sodium buffer, to prevent undesired interactions; moreover, the samples were not extruded. A Q2000 differential scanning calorimeter (Thermal Analysis Instruments, New Castle, DE) and TA Universal Analysis 2000 software were used to obtain measurements.

### Incorporation efficiency measurements

Parent compound and prodrug concentrations in liposomes were determined by UV-Vis spectrophotometry in triplicates (Absorption peaks at 2T-P: 292 nm; 2T-N: 285 nm). Prodrug-loaded liposomes were prepared at varying concentrations, where DPPC and DSPE-PEG2000 amounts remained fixed and prodrug amount varied from 0-50 mol%. Each sample was extruded through a 200 nm pore membrane for a total of 11 passes. Pre and post extrusion liposomes were ruptured by dissolution in DMSO (liposome suspension/DMSO, 1:9, v/v). Samples were analyzed in a quartz cuvette. The following parameters were used: scan speed: 120 nm/min; bandwidth: 2 nm; integration time: 0.15 sec; data interval: 0.30 nm; start wavelength: 500 nm; end wavelength: 190 nm.

### Liposomal particle size distribution and stability assay

Liposomal size distribution was determined with dynamic light scattering (DLS) using a Zetasizer Nano ZS90 (Malvern Instruments Ltd., Worcestershire, UK) equipped with the Zetasizer software. Measurements were taken with disposable polystyrene sizing cuvettes containing the liposome suspensions. Reported DLS measurements are averages of 3 individually prepared liposome samples.

### Cell culture

HeLa (human cervical cancer, ATCC S3) were cultured in DMEM (Invitrogen) supplemented with 10% FBS, 100 mg/L penicillin G, and 100 mg/L streptomycin. MCF-7 (human mammary carcinoma, ATCC HTB 22) cells were cultured in DMEM supplemented with 1.0 mM sodium pyruvate, 1% GlutaMax-1 (Invitrogen), 100 μg/mL penicillin, 100 μg/mL streptomycin, and 10% FBS. The cells were incubated at 37 ˚C in a humidified atmosphere with 5% CO_2_. MCF10A (human mammary epithelial, ATCC CRL10317) cells were cultured in RPMI (Invitrogen) supplemented with 5% FBS (Invitrogen), epidermal growth factor (20 ng/mL, QED Bioscience Inc.), hydrocortisone (0.5 μg/mL, Sigma), cholera toxin (100 ng/mL, Sigma), insulin (10 μg/mL, Sigma), and PenStrep (Invitrogen).

### Enzymatic assay

Enzymatic cleavage was qualitatively measured using a Shimadzu RF-5301pc Spectrofluorophotometer. Porcine liver esterase was diluted in 1X PBS from a concentrated stock solution to 1.2x10^-7^ M and stored at -20 ℃. At time zero, 100 μL of empty or prodrug-loaded liposomes were placed in a 100 μL cuvette and 5 μL of esterase was added. The solution was immediately measured in a Shimadzu RF-5301pc spectrofluorophotometer. The sample was measured again at 60 minutes. The following parameters were used: medium scanning speed, 2 second response time, 1 nm sampling interval, 3 nm excitation slit width, 20 nm emission slit width, high sensitivity, an excitation wavelength of 250 nm, and an emission range of 280 nm - 600 nm.

### *In vitro* cytotoxicity of prodrug-loaded liposomes

Cells were seeded at 4,000 cells/well (HeLa, MCF-7, MCF10A) in 96-well plates and incubated for 24 hours at 37 ^o^C and 5% CO_2_ to ensure cell adhesion. Following incubation, the media was replaced and the cells were divided in parallel into 2 treatment groups: prodrug-loaded liposomes and parent compound. The cells were treated with a two-fold dilution series of prodrug-loaded liposomes, beginning at either 2 vol% of the prodrug-loaded liposome suspension (prodrug/lipid, 20 mol%) or 1 µM of the parent compound. The prodrug-loaded liposome controls were as follows: negative controls (no treatment, empty liposomes (without drug, 2 vol%) and a positive control (phenylarsine oxide (PAO)). The parent compound groups controls were as follows: negative controls (no treatment, DMSO) and a positive control (PAO). After 48 hours of incubation, 20 µL of 3-(4,5-dimethylthiazol-2-yl)-2,5-diphenyltetrazoliumbromide (MTT, 5 mg/mL) was added to each well then incubated for 2 hours. Subsequently, 100 µL of DMSO replaced the media to dissolve the formazan crystals formed. Absorbance at 595 nm was measured via a ThermoMAX microplate reader. The experiments were performed in quadruplicate and repeated twice.

### Liposomal cellular uptake assay

MCF-7 and MCF-10A cells seeded in 6-well plates at 300,00 cells/well were incubated with 0, 5, or 20 mol% 2T-N and DiO (a fluorescent lipid dye) loaded liposomes at various concentrations for 24 h. After cellular uptake, cells were incubated with HoechstⓇ 33258 for 20 minutes then washed 3 times with 1X PBS to remove excess liposomes and dye. The intracellular fluorescence was observed using a Nikon Eclipse LV100 fluorescence microscope equipped with a Hamamatsu ORCA-RF camera. Image enhancing: Images were adjusted using ImageJ to improve visualization of assay results. A flat field correction was performed on bright field images to correct for pre-existing artifacts. Image brightness and contrast were equally enhanced for the purpose of display and publication.

### Transmission electron microscopy

Tetramethylorthosilicate (TMOS) was deposited for 15 min onto a 20 mol% 2T-N liposome sample creating silica coated vehicles using the Sol-Generating Chemical Vapor into Liquid deposition method discussed in Johnston 2017 38. A drop of silica-coated liposomes was absorbed onto a holey carbon coated 200-mesh copper grid and excess was removed using filter paper. After the samples dried at room temperatures liposomes were imaged using a transmission electron microscopy (model JEOL 2010F; University of New Mexico, Albuquerque, NM).

### Microbubble preparation

Microbubble prodrug-loaded lipid films were produced via premixed lipid chloroform solutions of 1,2-dipalmitoly -sn- glycero-3-phosphocholine (DPPC); 1,2-dipalmitoyl -sn- glycero -3-phophate (monosodium salt) (DPPA); 1,2-distearoyl -sn- glycero-3-phosphoethanolamine -N-(polyethylene glycol) -5000) (ammonium salt) (DSPE-PEG5000) mixed with the prodrug solution in chloroform at the desired lipid ratio [DPPC: DSPE-PEG5000: prodrug]. The lipid mixture was then dried under nitrogen gas and further under vacuum at 50 ◦C for 2 h. The prodrug-enriched lipid film was resuspended in 1.5 mL aliquots of (80 vol% 0.1 M Tris, 10 vol% glycerin, 10 vol% propylene glycol) Tris buffer via sonication bath for 30 min at 50 ◦C resulting in a 3 mg/mL of Tris buffer creating a liposome suspension. Post sealing, each vial was purged with 10mL of sulfur hexafluoride (SF_6_). A Vialmix (mechanical shaker) was used to shake the vials for 45 seconds to form microbubbles from the liposome suspension 3.

### Nanodroplet preparation

Preformed microbubbles [DSPC: DSPE-PEG2000] with perfluorobutane were used to generate nanodroplets following the procedure presented in Dayton 2011 8. The microbubble containing vial was submerged in a 5 ^o^C CO_2_/isopropanol bath and vented with a 27 G needle then pressurized with approximately 30 mL of air (from room).

### Localized delivery to adherent cells with ultrasound

Microbubbles generated with 0 mol% and 20 mol% prodrug concentrations were purified by centrifugation at 0.3 rcf for 10 mins, separating the supernatant (microbubbles) and infranatent (liposomes). The supernatant was incubated in culture media with serum for 12 hrs, then centrifuged again. HeLa cells were seeded at 120,000 cells per well onto thermanox coverslips in 6-well plates. Once confluent cells were observed, coverslips were setup into the cell-plate chamber in contact with 2 μL microbubbles: 3 mL media. Chambers were then sealed with another thermanox coverslip, placed in the ultrasound chamber. and exposed to 18 pulses of ultrasound. They were immediately introduced to 3 washes of media to remove excess microbubbles 39, 40. After 20 hours of incubation, cell plates were imaged in bright field using a Nikon Eclipse LV100 fluorescence microscope equipped with Hamamatsu ORCA-RF camera and Nikon Plan Fluor objective lens. Images were cropped and brightness/contrast was equally enhanced for the purpose of display and publication. Controls were no treatment and inverted cell-plates to facilitate contact with 0 mol% and 20 mol% prodrug-loaded microbubbles following the protocol above, except without ultrasound exposure.

### Simulation model preparation

The linker and prodrug moieties and the drug molecules were modeled using atom types from the Lipid14 force field, where available, and otherwise from the General Amber Force Field (GAFF) 41, 42. Partial point charges were calculated using the restrained electrostatic potential (RESP) method 43 with the Hartree-Fock 6-31G* basis set. Gaussian 09 software was used for the charge calculations, with preparatory assistance from the R.E.D. Server and R.E.D. III software 44, 45. For the prodrugs and linker, a fixed-charge “capping” strategy was developed based on the Lipid11 modular framework approach 46. A “head cap” was attached to each prodrug moiety during the charge fitting and later removed. The linker moiety was charge-fitted with a “tail cap” on the end to be joined to the prodrug and a “head cap” on each end to be joined to the fatty acyl tails; all caps were removed after charge-fitting. Each prodrug was joined to a linker moiety using the AmberTools preparatory program LEaP 47, and the resulting prodrug-linker construct was treated as a “residue” for further simulation system construction.

Hydrated lipid bilayer systems were prepared using the CHARMM-GUI membrane builder, followed by the introduction of drug or prodrug molecules through targeted molecular substitution 48. Table 1, provides the detailed molecular composition of each simulation system. Every system used 1,2-dipalmitoyl-*sn*-glycero-3-phosphocholine (DPPC) as the base lipid and included approximately 35 TIP3P water molecules 49 per lipid—or 50 water molecules for the systems incorporating 2T-prodrug. K^+^ ions were used to neutralize the net negative charge of the 2T-prodrug molecules and were added to the aqueous layer by replacing water molecules. In all systems, the number of K^+^ counter ions equaled the number of 2T-prodrug molecules (if any).

### Simulation details

CUDA-enabled Amber 14 biomolecular simulation software was used to conduct all-atom, tensionless molecular dynamics simulations 47, 50. Every system was simulated in duplicate, giving two independent trajectories, each with 150 ns of production (the portion used for analysis). For the 2 mol% drug and prodrug systems, all production runs were extended to 300 ns, to facilitate ample sampling of the potential energy landscape. For all systems, the temperature was held constant at 323 K (50°C)—above the phase transition temperature for DPPC—using Langevin temperature control with a collision frequency of 1.0 ps^-1^. The pressure was maintained at 1.0 bar, using the Berendsen barostat 51. The SHAKE algorithm was used to constrain bonds to hydrogen, with a tolerance of 10^-7^ Å, and the simulation timestep was set to 1.0 fs. Particle mesh Ewald was used to calculate nonbonding interactions 52, with a cutoff distance of 10 Å. Translational center-of-mass motion was removed every 2 ps, and coordinate wrapping was turned on.

Atomic positional histograms were calculated at 1-Å intervals along the bilayer normal (*z*-axis) using the AmberTools program CPPTRAJ 53. The locations of the atoms used to generate the histograms are indicated with arrows in Figure S9. For plotting, the histograms were averaged across the duplicate trajectories for each system. Carbon-wise lipid tail order parameters, S, were calculated with CPPTRAJ. The order parameters for carbons 4-8 were averaged to generate a “plateau” value, 〈S〉, for ready comparison across simulation systems. Common rules of error propagation were applied to estimate standard deviations for the 〈S〉 values, based on the standard deviations reported by CPPTRAJ. Simulation snapshot images were rendered with PyMOL 54.

## Results and Discussion

### Structural validation and synthetic yield of prodrugs

**Figure 2:**
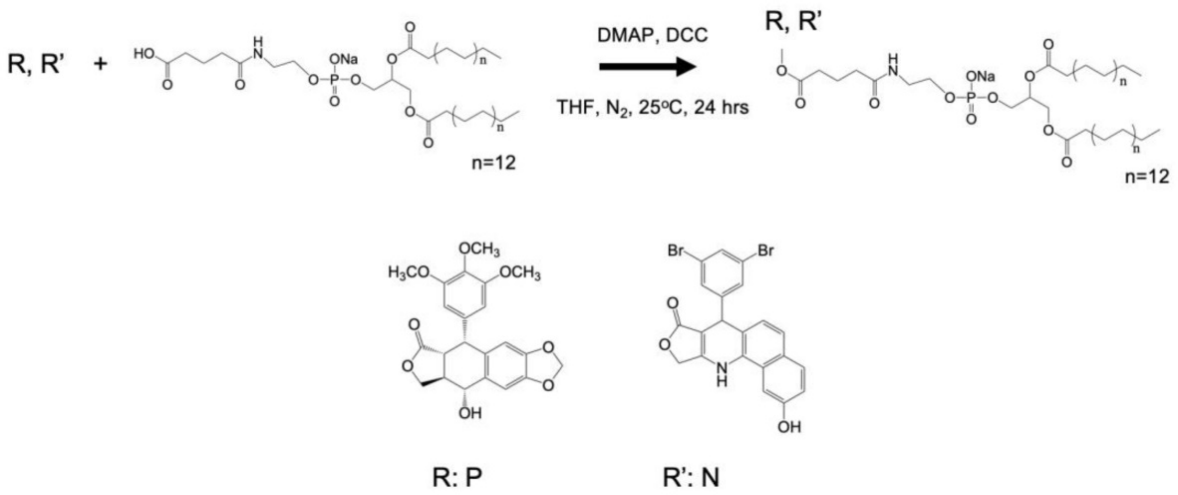
Steglich esterification of chemotherapeutic compounds with DPPE-Glu.

**Figure 3:**
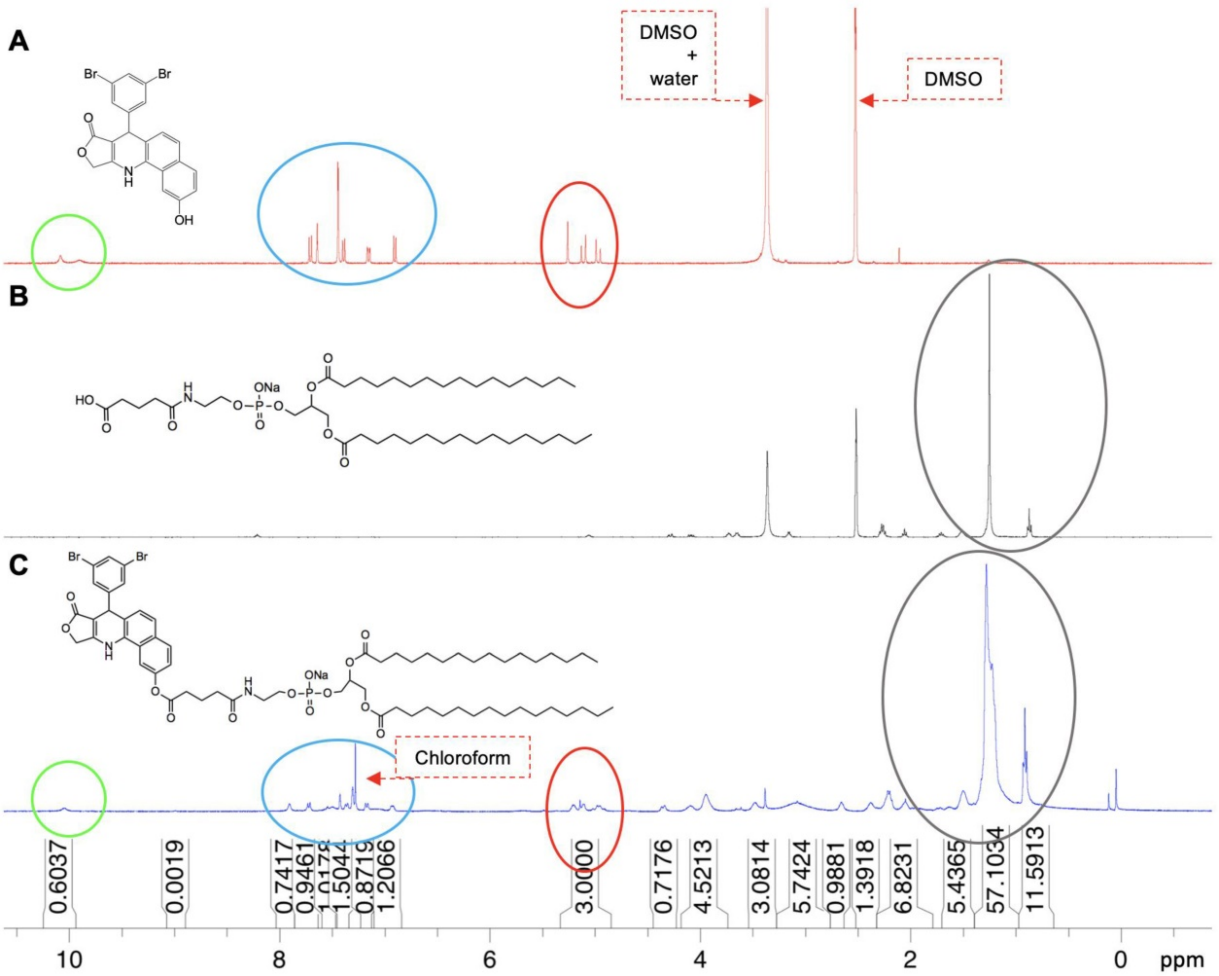
H^1^ NMR spectra of **(A)** N, **(B)** DPPE-Glu, and **(C)** 2T-N.

Phospholipid chemotherapeutic prodrugs, 2T-P and 2T-N, were synthesized for use in lipid-based carriers for drug delivery with ultrasound, Figure 1. Utilizing a Steglich reaction, P and N were coupled to DPPE-Glu to produce the final prodrugs, 2T-P and 2T-N, Figure 2. Structures were verified with ^1^H, ^13^C, and high resolution mass spectrometry as shown in Figures S1-S8. Figure 3 shows a representative set of ^1^H NMR spectra confirming successful conjugation of 2T-N. The parent compound N, Figure 3A, peaks located around 4.99-5.13 ppm correspond to the amine group's signal. DPPE-Glu (Figure 3B) gave signals in the range 0.89-1.31 ppm, corresponding to protons of -(CH_2_)-. All of the characteristic peaks appeared in the 2T-N spectrum, Figure 3C, and an increase in the carbonyl signal at 2.2 ppm further validated the conjugation between DPPE-Glu and N. The co-existent peaks related to the protons on N and DPPE-Glu observed in the detailed ^1^H NMR spectrum confirm the formation of 2T-N (CDCl_3_**-** *d*_6_, d, ppm): 10.56 (s, 1H), 8.06 (s, 1H), 7.89 (d, *J*=6.88 Hz, 1H); 7.72 (d, *J*=8.96 Hz, 1H), 7.59 (s, 1H), 7.44 (s, 1H), 7.37-7.29 (m, 3H), 7.19 (d, *J*=8.8 Hz, 1H), 6.96 (d, *J*=8.36, 1H), 6.38 (d, *J*=7.08 Hz, 1H), 5.25 (s, 1H), 5.13-4.99 (m, 3H), 4.34 (d, *J*= , 1H), 4.02-4.01 (m, 5H), 3.51 (d, *J*=14.08 Hz, 2H), 3.05 (s, 3H), 2.69 (s, 2H), 2.41 (s, 2H), 2.23 (d, *J*=6Hz, 4H), 2.09 (s, 2H), 1.31-1.21 (m, 46H, -C_14_H_46_) 0.91-0.89 (m, 6H).

The one-pot synthesis of 2T-P and 2T-N resulted in low yields, 28.6% and 25.6%, respectively, which may be attributed to the following possible causes: 1) low formation of the intermediate (O-(uridine acetonide)-succinoyl-isourea) reduced the reaction rate; 2) the bulky stature of P, N, and DPPE-Glu may generate steric effects, with DCC decreasing successful catalyst collisions with the intermediate; 3) high formation of side products 55.

### Prepared Vehicles

#### DSC thermograms illustrate that prodrugs intercalate within bilayer, while parent compounds are excluded

**Figure 4:**
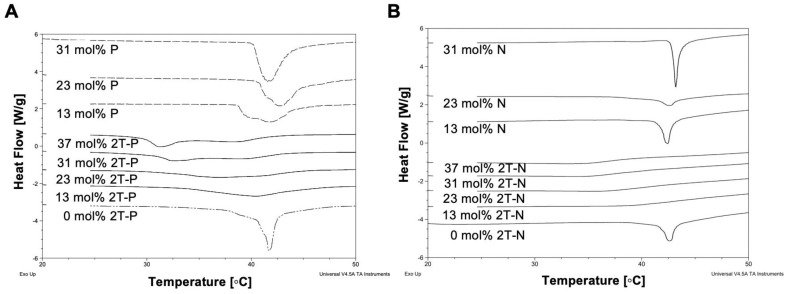
Differential scanning calorimetry (DSC) curves of **(A)** 2T-P, P, **(B)** 2T-N, and N loaded liposomes with increasing concentrations.

**Figure 5:**
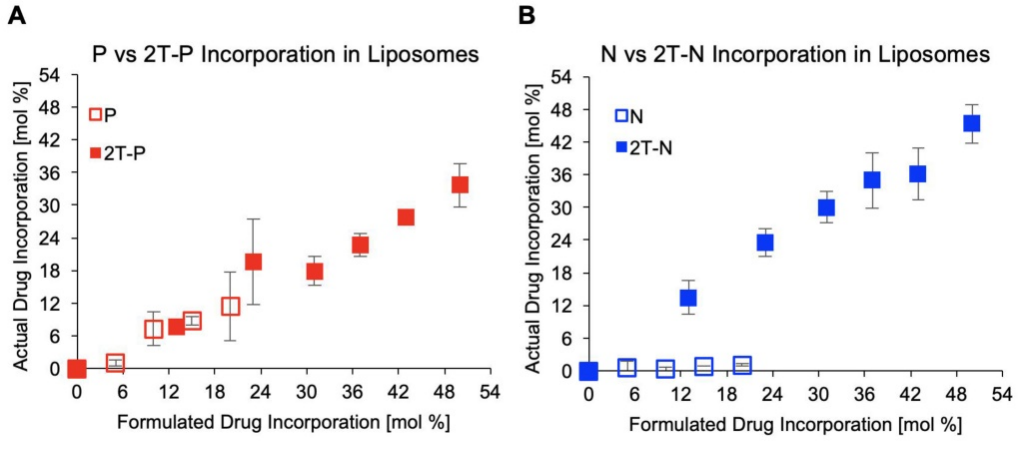
Drug incorporation within liposomes post extrusion, n=3.

DSC can be used to study liposomal drug delivery systems by determining the compatibility of mixed molecules and compositions within the liposome bilayer 56, 57. This is done by observing the thermotropic behavior of the liposomes at varying prodrug concentrations, to see whether insertion of these molecules modifies the phase transition temperature 57, 58, Figure 4. A sharp endothermic peak with a pre-phase transition is characteristic of DPPC bilayers, but in this case the pre-phase transition is masked by DSPE-PEG-2000 59. In comparison to empty liposomes, prodrug-loaded liposome (2T-P and 2T-N; 0 to 37 mol%) thermograms show shallow decreasing phase transition temperatures with increasing prodrug compositions 57, 60. In contrast, the thermograms of P and N loaded liposomes (0 to 31 mol%) did not show a significant change in the phase transition temperature and the magnitude of the endothermic peak was modulated as a function of loading. The decrease in the 2T-P and 2T-N loaded liposome endothermic peak suggests a *change* in lipid-lipid interaction, as a result of the prodrug presence in the bilayer 57. P induced a slight change in the phase transition at 13 mol%, corresponding to drug saturation (Figure 5). However, as the concentration increased, the phase transition returned to that of 'empty liposomes'. This suggests that at a higher concentration, P is excluded rather than becoming entrapped in the bilayer 56, 61. According to incorporation data (Figure 5), N did not reduce the phase transition temperature, whereas 2T-P and 2T-N did.

#### Lipid conjugation increases incorporation within liposomes

N and P are both hydrophobic compounds that have been tethered to a phospholipid moiety through a cleavable linker moiety (2T-P and 2T-N), increasing their incorporation within liposomes, as characterized by UV-vis spectrometry. A series of parent compound and prodrug-loaded liposomes concentrations, ranging 0 to 50 mol%, were formulated then extruded through a membrane to remove unincorporated precipitates. Samples were analyzed by measuring the absorption peaks for 2T-P and 2T-N around 280-300 nm pre- and post- extrusion, by rupturing the liposomes via dissolution in DMSO to compare loading capacities. As anticipated, the maximum loading for P (~11 mol%) and N (~1.1 mol%) were low, whereas 2T-P (36 mol%) and 2T-N (46 mol%) were considerably higher, Figure 5. Both prodrugs attained high loading capacities with 2T-N achieving a 96% loading efficiency. Samples prepared beyond 50 mol% incorporation were not tested due to difficulty in extrusion. It is important to have high prodrug incorporation through self-assembly, to increase the loading efficiency and reduce purification steps for the clinical preparation of liposomal formulations 22, 62, 63. Achieving high loading efficiencies are desirable as there is a high therapeutic compound-to-lipid mass ratio, reducing the number of liposomes needed to be administered to attain the necessary therapeutic effect. For these prodrugs, the lipid moiety is a member of the bilayer, which is also beneficial for use in lipid monolayer vehicles like microbubbles and nanodroplets 64-67. These prodrug structures result in a higher drug loading limit and increased stability than unmodified agents as previously reported 37, 58, 61, 68.

#### Prodrug-loaded liposomes remain stable over time

**Figure 6:**
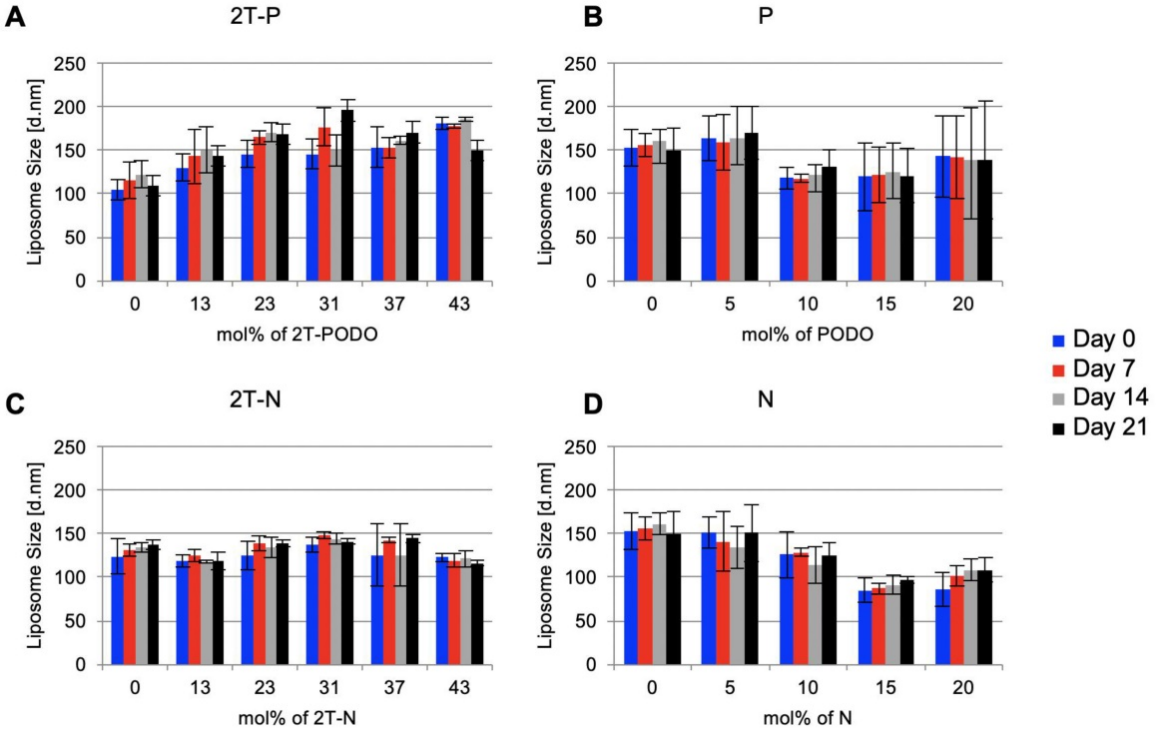
Drug loaded liposome stability assessed by size distribution of the liposomes over 3 weeks using dynamic light scattering, n=3.

The effect of prodrug concentration on prodrug-loaded liposomes physical stability was assessed using dynamic light scattering. If there is minimal change in the size distribution of the liposomes over time, it is presumed that the liposomes maintain physical stability 69, 70. Measuring the size distribution of prodrug-loaded liposomes over time showed 2T-P and 2T-N-loaded liposomes remained nearly constant in size throughout a 3-week period, as shown in Figure 6A and 6C. Extruded P and N loaded liposomes, with maximal incorporations of 1 and 11 mol%, respectively (concentration labels in Figure 6 reflect the formulated drug amount rather than the final drug retention post extrusion), remained stable for 3 weeks, Figure 6B and 6D. Due to the consistency of the prodrug-loaded liposome size distributions and visual observations, it is speculated the prodrugs remain within the liposomes without precipitation. Full saturation was assumed when extrusion became impossible to complete along with the observation of a cloudy suspension, indicating unincorporated drug particles were present.

Our results suggests increasing both the loading efficiency and encapsulation stability minimizes chemical degradation and allows for a feasible prodrug formulation in lipophilic vehicles while maintaining a spherical morphology, Figure 1 71. Overall, solubility of P and N was increased by conjugation to phospholipids, converting the hydrophobic drug into a lipophilic prodrug for improved biodistribution and pharmacokinetics 61, 71, 72. This alteration allows for high entrapment, low release in storage media, and high stability 61. Successful application of this approach could result in reduced treatment schedule or flexible dosing dependent on the treatment strategy. The stability of prodrug-loaded liposomes shows promise for reasonable shelf life when compared with clinically used liposomes and potential for lyophilization for storage 73, 74. This prodrug strategy has potential to be implemented not only for chemotherapeutic applications, but also for antiviral, antibacterial, and antifungal treatments. Previously unusable drugs, most often due to solubility toxicity, may be altered for formulation in targeted lipid-based carriers.

### Simulation Results

#### Simulations provide physical insight into compound incorporation

Atomistic molecular dynamics simulations of parent compound (P and N) and prodrug (2T-P and 2T-N) incorporation in model liposomes were conducted at various loading levels. The objectives were to visualize modes of incorporation, to determine positional preferences within a lipid-based carrier, and to provide atomic resolution insight into the effect each compound has on the vehicle structure. The lipid-based carrier was modeled as a lipid bilayer. Each compound was incorporated within a bilayer of DPPC lipid, at levels ranging from very low (2 mol%, or 1 molecule per bilayer leaflet), to excessively high (20 mol% for P and N; 60 mol% for 2T-P and 2T-N). Compositional details are given in Table 1.

The simulations used atom types from the generally well-validated General Amber Force Field (GAFF) and Lipid14 force field 41, 42. Control DPPC bilayers showed good agreement with experimental values for order parameter and area per lipid (Table 1), as expected for the Lipid14 force field 41. In agreement with our DSC data (Figure 4), the simulations indicate that P, N, 2T-P, or 2T-N compound incorporation alters the lipid bilayer physical properties in a composition-dependent manner. Though qualitative, this comparison provides cross-validation of the DSC and simulation techniques used in this study. Details of the physical changes upon compound incorporation are discussed below.

#### Flexible, headgroup-like linker facilitates compound incorporation within and just outside the bilayer

**Figure 7:**
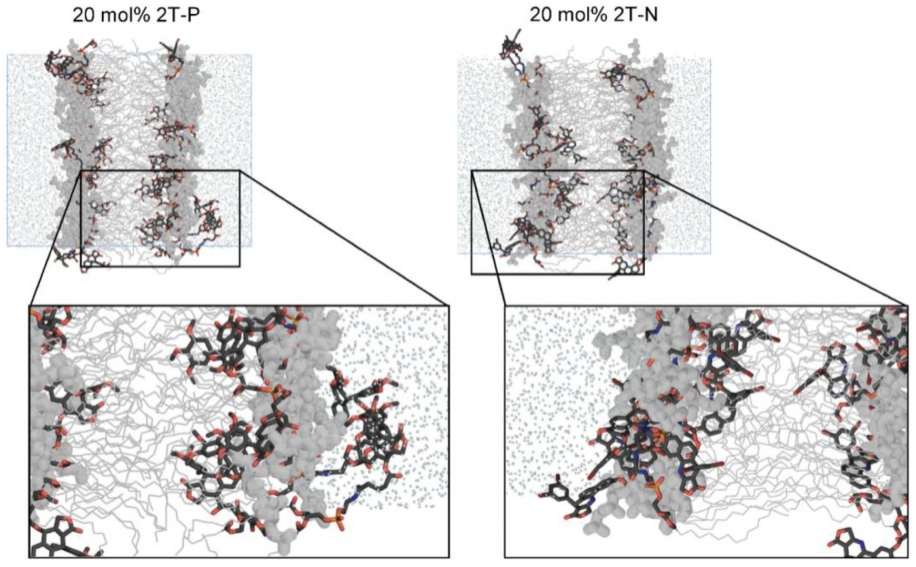
Simulation images of systems incorporating 20 mol% 2T-P or 2T-N. DPPC lipids shown in gray, with tails depicted as thin lines and headgroups as thick lines. Explicit water molecules shown as reduced-size dots. All H atoms hidden for clarity. Drug and linker moieties colored by atom, as follows. black C, red O, orange P, and blue N.

**Figure 8:**
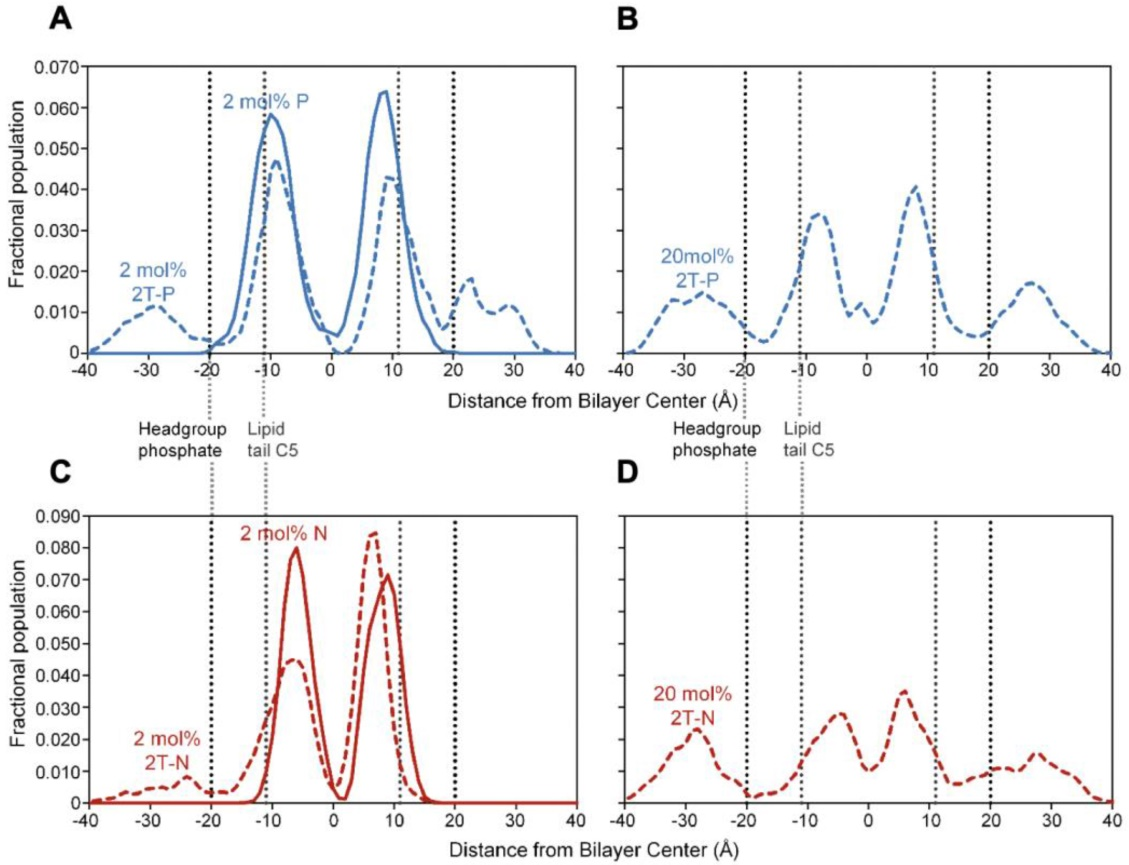
Positional histograms for a terminal atom on the drug molecule (central methyl carbon for P and 2T-P and bromine for N and 2T-N), at incorporation levels of 2 mol% **(A and C)** or 20 mol% **(B and D)**. Dotted lines indicate the positions of the headgroup phosphate moiety and the 5th carbon of the lipid tail (C5), together delineating the polar headgroup region on either side of the bilayer.

Figure 7 shows simulation snapshot images of bilayers incorporating the 2T-P and 2T-N compounds, and Figure 8 provides histograms of compound locations relative to the bilayer center. Simulations at the minimal incorporation level of 2 mol% indicate that all of the parent compounds and prodrug moieties preferentially localize to the bilayer interior (Figure 8A and C). N and 2T-N are found almost exclusively within the hydrophobic tail region, as delineated by the average position of the 5^th^ tail carbon atom (C5, dotted line near ±11 Å bilayer depth in Figure 8C). P and 2T-P have broader positional distributions, spreading into the headgroup region located between the two dashed lines near ±20 and ±11 Å bilayer depth (Figure 8A). 2T-P has secondary populations in the aqueous region just outside the bilayer (±40 to ±20 Å) at 2 mol% and above (Figure 8A and B and data not shown). Similar “aqueous populations” occur for 2T-N at and above the higher prodrug incorporation level of 20 mol% (Figure 8D and data not shown). Similar concentration-dependent localization effects have been observed with the bulky electron-spin-resonance probe tempocholine, which prefers burial in the lipid bilayer tail region at lower incorporation levels (2 or 11 mol%) and develops a secondary population in the water-bilayer interfacial region at a higher incorporation level (28 mol%) 75, 76.

**Figure 9:**
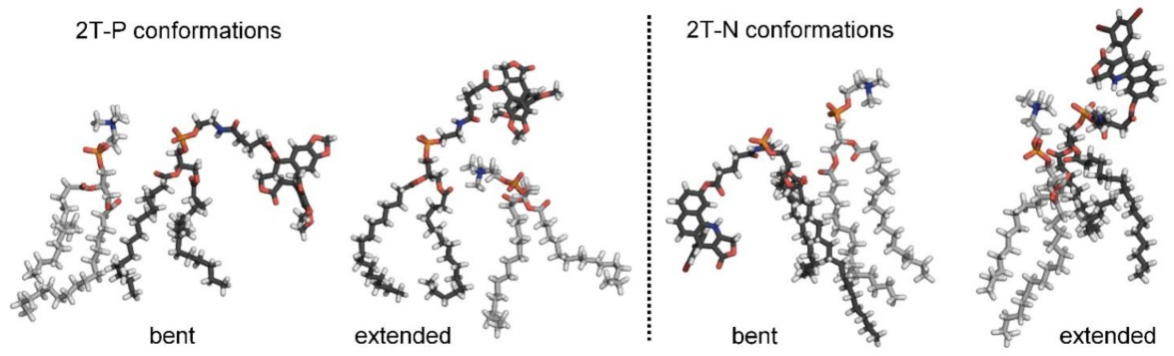
Bent and extended 2T-P and 2T-N prodrug conformations, as observed in simulations at 20 mol% incorporation levels. Prodrug molecules are shown with black carbon atoms; DPPC molecules shown with gray carbon atoms. Other atoms colored as in Figure 7, with H atoms in white.

In the current work, the prodrug populations inside the bilayer are facilitated by “bent” conformations of 2T-P and 2T-N, while the aqueous populations are supported by “extended” conformations (Figure 9). This, too, is in agreement with simulations of tempocholine 76. The flexible linker used in our study supports drug loading by enabling both bent and extended conformations to occur, while maintaining tail-tail contacts between the prodrug molecules and phospholipids in the hydrophobic region.

Besides being flexible, the linker moiety is headgroup-like in its structure (Figure 2 and 9). It is, thus, able to replace some headgroup-headgroup ionic interactions with ionic and ion-dipole interactions via the linker's phosphate and amide NH groups. Table 1 includes the total number of polar contacts (ionic and ion-dipole) observed in simulations for the various compound incorporation levels. The number of headgroup-headgroup polar contacts is reduced in direct proportion to the number of P parent compound molecules substituted for DPPC lipids. The N parent compound shows somewhat less reduction in the number of polar contacts, perhaps because it buries deeper in the bilayer than P and, thus, has less impact on the headgroup region structure. The lipid conjugates have comparably little impact on the number of headgroup-headgroup and headgroup-linker contacts, maintaining polar contact counts close to 90% of the total number possible (i.e., 2 polar groups per DPPC lipid, divided by 2 molecules needed to form a contact, giving 1 contact per lipid or lipid prodrug), even at 45 mol% prodrug incorporation. In addition, the systems incorporating 2T-P or 2T-N prodrug are resistant to change in area per lipid, giving fairly stable values at loading levels up to 45 mol%. Lipid order parameter does increase, reflecting increased packing of molecules in the lipid tail region. The lipid order parameter value peaks at 0.216 for 20 mol% 2T-P (7% higher order than DPPC) and at 0.228 for 20 and 45 mol% 2T-N (13% higher than DPPC).

#### Excessive amounts of parent and prodrug compounds physically disrupt the bilayer

Our simulations indicate that at high incorporation levels (10-20 mol%), the P and N parent compounds disrupt the bilayer, leading to increased order parameter and reduced area per lipid (entropically unfavorable), along with reduced polar contacts (enthalpically unfavorable). Much higher 2T-prodrug incorporation levels (>45 mol%) likewise lead to bilayer structural disruption. At these levels, physical changes include reduced lipid order parameter—especially relative to the increased order parameters for the 2T compounds at 20 and 45 mol%—along with substantially increased area per lipid (~70 Å^2^ *vs*. 62 Å^2^ for DPPC) and strongly reduced polar contacts (<80% of the total number possible). Together, these changes agree with our observations that parent compound levels above 10 mol% and lipid prodrug incorporation levels above 45 mol% were not feasible in the laboratory.

It is somewhat unclear from the simulation data why the N parent compound did not incorporate experimentally at levels above 5 mol%. However, we do observe a larger increase in order parameter and decrease in area per lipid for N at 10 mol%, compared with P at the same incorporation level: the order parameter increases to 15% above the DPPC control for 10 mol% N but only 6% above DPPC for 10 mol% P. These data suggest that the N parent compound incorporation is entropically disfavored to a greater extent than P parent compound incorporation. The stronger impact of N, relative to P, on lipid order is also seen with the 2T-prodrug compounds at 20 and 45 mol% incorporation.

Compound incorporation efficiency within a lipid-based carrier reflects a balance among molecular geometry, order/disorder, and favorable interactions. Minimal disruption of the vehicle is essential, especially for microbubble lipid-based carriers. Vehicle stability is conferred by the hydrophobic effect (exclusion of nonpolar constituents from water), the large number of favorable van der Waals interactions among the lipid-based carrier hydrocarbons, and favorable headgroup-headgroup ionic interactions. The two-tailed-linker construct used as a prodrug anchor in this work achieves minimal disruption of the vehicle by mimicking the DPPC tails and replacing some of the headgroup-headgroup contacts. It also maximizes drug loading, by enabling hydrophobic drugs to be accommodated both in the nonpolar tail region and at the vehicle/water interface.

We anticipate that this construct will also be useful for delivering hydrophilic compounds (e.g., highly toxic chemotherapeutics), but somewhat reduced incorporation efficiency for such compounds is likely because their polarity will hinder localization in the hydrophobic lipid-based carrier interior. Based on the results shown here, we hypothesize that hydrophobic compound incorporation is limited by the size of the compound, the ability of the lipid-based carrier to generate “space” for it in the hydrophobic interior, plus the amount that can be placed at the water-bilayer interface. For the size of drug studied here, it appears that ~45 mol% is the maximum possible loading level when a two-tailed anchor is used. Future exploration of 4-tailed (e.g., cardiolipin) variants may be useful for expanding the size and/or amount of compound that can be incorporated.

### *In-Vitro* Experiments

#### Liposome cellular uptake

**Figure 10:**
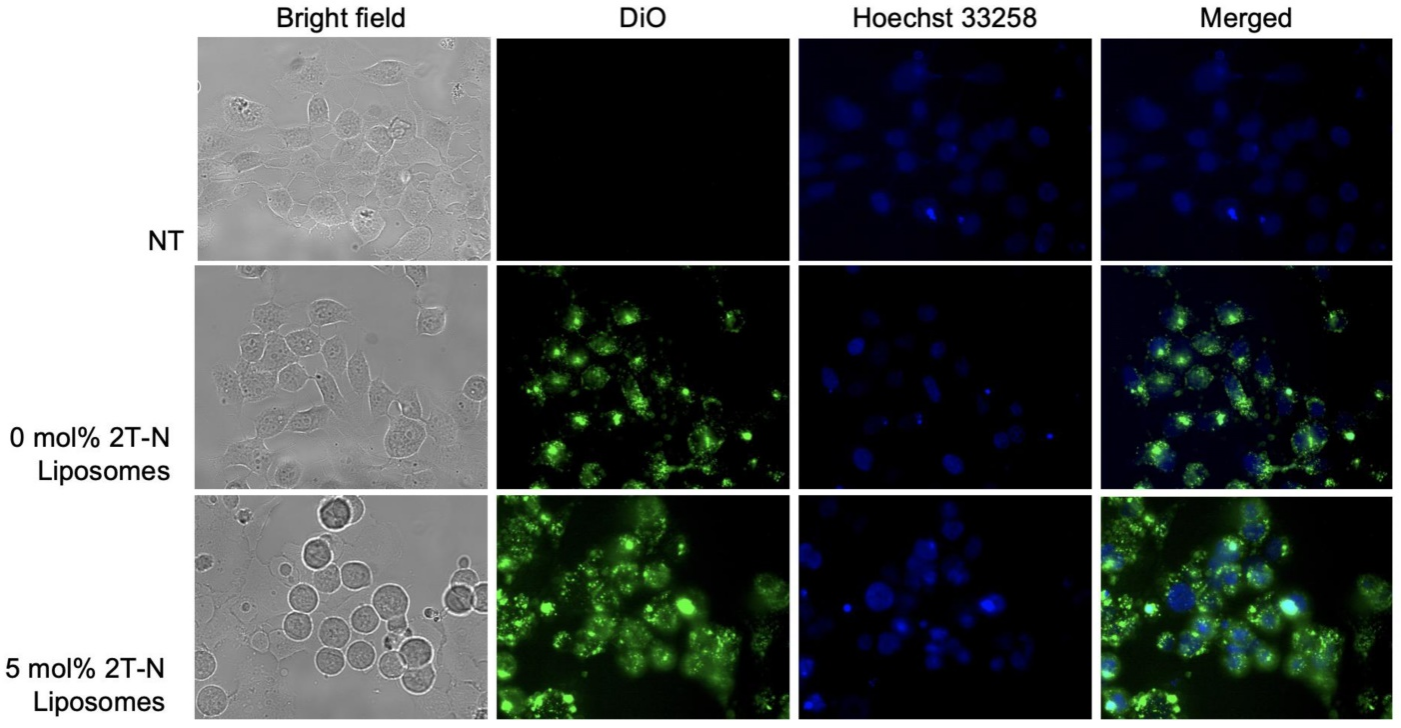
Cellular uptake in MCF-7 cells post 24 hour incubation with DiO and 2T-N loaded liposomes at a 0.7 μM concentration.

To ensure the cytotoxic activity is attributed to the prodrug incorporated liposomes, a cellular uptake assay was implemented. DiO was used to visualize the liposomes. MCF7 and MCF10A cells successfully internalized 0, 5, and 20 mol% 2T-N liposomes loaded with DiO, Figures 10 and S12. At 24 hours, Figures 10 and S12 show cells treated with 0 mol% remained attached with a healthy morphology post incubation, whereas cells treated with 2T-N result in a balled-morphology suggesting apoptosis.

#### 2T-N loaded liposomes retain N activity

The anti-proliferative activities of the prodrug-loaded liposomes (20 mol%) and parent compounds were examined in HeLa (cervical cancer), MCF7 (breast cancer), and MCF10A (noncancerous breast tissue) cell lines via 48 hr MTT assays. 2T-N prodrug-loaded liposomes maintained parent compound (N) potency, whereas with 2T-P loaded liposomes, activity was greatly reduced. 2T-P activity decreased by 3 orders of magnitude in HeLa and MCF7 cell lines compared with parent compound, while 2T-N maintained activity within 1 order of magnitude in HeLa and MCF7 cells (0.020 µM and 0.038 µM, respectively). These findings are important as it is essential for the prodrug to retain its activity. Additionally, a degree of selectivity was observed: 2T-N activity decreased by 3 orders of magnitude in noncancerous MCF10A lines (1.105 µM). The concentration (20 mol%) in the formulation was chosen below the saturation point in the lipid bilayer (to maintain stable liposomes). The cytotoxicity time was originally 96 hours to discern whether a delayed 'release' time was responsible for 2T-P's reduced activity (data not shown). Activity was not retained leading to the hypothesis that steric hindrance prevents cleavage at the intended site by intracellular enzymes.

While ultrasound will spatially enhance delivery, enzymatic activation after intracellular uptake is still required, likely within lysosomal and endosomal compartments. The concentration of various enzymes is increased in certain cancers, such as elevated phosphofructokinase in HeLa cells and pancreatic cancers with increased secreted phospholipase A2 (sPLA2) 77-79, which may provide an additional advantage. However, the 2T-N prodrug enzyme specificity is currently unknown. Preliminary nonspecific enzymatic assays with dynamic light scattering and fluorescence spectrophotometry results are shown in Figure S10 and S11, where liposomes containing 2T-N and incubated with esterase result in a slightly modified size distribution, polydispersity index, and the appearance of a 20-40 nm subpopulation. The fluorescence spectra were also monitored upon addition of esterase resulting in decreased intensity of 2T-N containing liposomes and an increase in intensity for empty liposomes, suggestive of enzyme driven changes. We hypothesized that porcine liver esterase (nonspecific) hydrolyzes the ester bonds and activates the prodrug resulting in the cytotoxicity observed. HPLC or LC-MS is needed to confirm the structure of the degradation products. If cleavage occurs at a more accessible bond such that the parent compound structure is not regained, the function and therefore biological activity may be significantly reduced 34. Without nanomolar toxicity within 72 hours, the 2T-P prodrug may have limited applications.

#### Ultrasound localized delivery of 2T-N microbubbles

**Figure 11:**
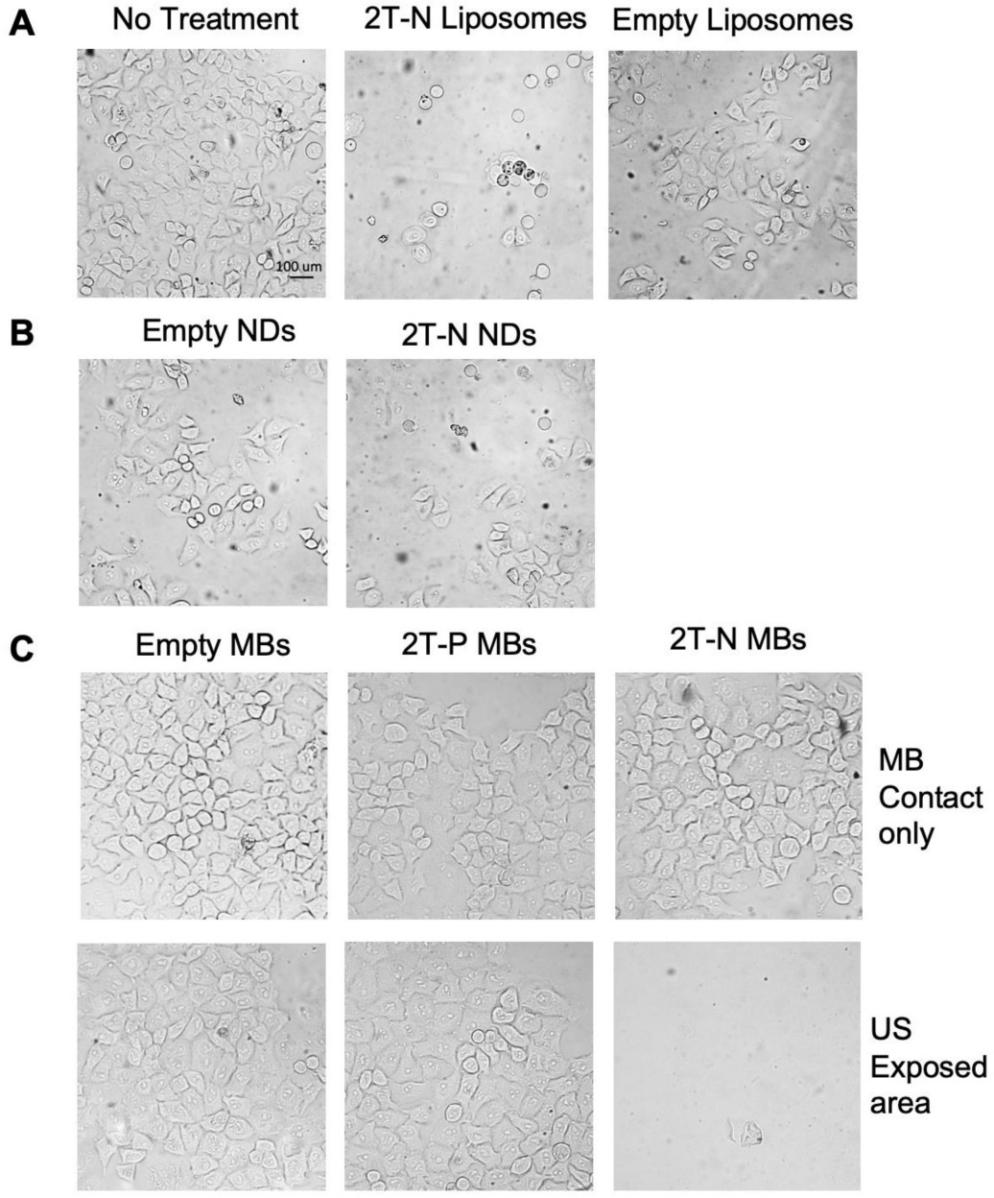
*In vitro* prodrug loaded vehicle delivery. **(A)** Prodrug loaded liposome contact only, **(B)** ultrasound triggered nanodroplet delivery, **(C)** With and without ultrasound triggered delivery of prodrug loaded microbubbles.

After establishing that 2T-N retained potency in liposomes, we performed a proof of concept experiment using microbubbles and phase changing nanodroplets, both incorporating the 2T-N prodrug, for focused drug release with ultrasound. *In vitro* cytotoxicity of localized microbubble and nanodroplet delivery via ultrasound studies validate triggered delivery with 2T-N loaded microbubbles and nanodroplets. Figure 11B and 11C displays a representative image set from triplicate results using prodrug-loaded microbubbles and nanodroplets. Microbubble dissociation was not observed during incubation with serum, suggesting they will remain intact upon intravenous injection. Empty microbubbles and prodrug-loaded microbubbles were placed in contact with HeLa cells for ultrasound free controls to confirm localization via ultrasound, Figure 10C left panel. Empty and 2T-P microbubble ultrasound treated cells remained confluent in the ultrasound exposed and unexposed areas, whereas 2T-N microbubble ultrasound treated cells diminished in the ultrasound exposed area but remained confluent in the ultrasound unexposed areas. Similarly, empty and 2T-N loaded nanodroplets exposed to ultrasound showed a reduction of cells in the targeted area, Figure 11B. Nanodroplet drug delivery vehicles have advantages of both liposomes and microbubbles. It is expected that nanodroplets circulate throughout the vascular system upon injection like liposomes, but expand into microbubbles upon ultrasound exposure for targeted and real-time deposition. Nanodroplets also have a longer half-life in blood than microbubbles, increasing the opportunity for the nanodroplet to flow through the target vasculature.

This study demonstrates that conjugating a potent drug to a phospholipid tail can achieve high loading efficiency, stability, retention, and targeted delivery *in vitro*. The strategy employed here has achieved both physical and chemical means of targeting. An extracorporeal ultrasound application, such as sonoporation, can mechanically aid drug delivery through difficult areas, such as the stroma 17. Currently, stroma reducing strategies combined with systemic chemotherapy have yet to positively impact patient tumors 80, 81. Enzymatic degradation of the prodrug can target organs with selectivity towards cancerous cells, avoiding unwanted burst release. These delivery strategies with lipophilic vehicles keep the drugs 'hidden,' providing a way to avoid the body from building resistance as the vehicles may bypass the efflux pumps. Currently, we have chosen to apply this linker strategy to topotecan, cytarabine, and gemcitabine (pancreatic chemotherapeutics) due to their steric appeal 82. Furthermore, clinically used hydrophobic and hydrophillic combination therapies induce synergistic effects while reducing drug resistance. Applying these lipid prodrugs into a combination technique, such as done by resveratrol and paclitaxel, conveys potential in overcoming loading complications 83, 84.

## Conclusion

In these studies, we demonstrate the characterization and utilization of novel lipid conjugated prodrugs within lipid drug delivery vehicles for theranostic applications with ultrasound. This conjugation strategy could potentially be applied to an array of compounds and lipid vehicles, including irinotecan, topotecan, cytarabine, and gemcitabine, making them more appealing candidates for clinical treatment limiting off target effects.

## Supplementary Material

Supplementary figures and tables.

## Figures and Tables

**Table 1:** Physical data calculated from simulations

System		# DPPC^*a*^	# compound^*a*^	Order parameter^*b*^	Area per lipid (Å^2^)^*c*^	Polar contacts*^ d^*
DPPC simulation experiment^*e*^		128	—	0.202	62	125 (98%)
		—	—	0.198	63 ± 1	—
2 mol%	P	126	2	0.202	62	121 (96%)
	N			0.205	62	123 (98%)
10 mol%	P	116	12	0.214	59	105 (91%)
	N			**0.233**	**57**	111 (96%)
20 mol%	P	102	26	0.215	**56**	**81 (79%)**
	N			**0.253**	**53**	87 (85%)
2 mol%	2T- P	126	2	0.200	63	123 (96%)
	2T- N			0.200	63	123 (96%)
20 mol%	2T- P	102	26	0.216	66	115 (90%)
	2T- N			**0.228**	64	120 (94%)
45 mol%	2T- P	70	58	0.212	65	116 (91%)
	2T- N			**0.228**	64	113 (88%)
60 mol%	2T- P	52	76	**0.196**	**69**	**101 (79%)**
	2T- N			**0.196**	**70**	**99 (77%)**

Shading indicates unphysical systems—i.e., incorporation levels inaccessible in the laboratory. Substantial changes in **bold type**. *^a^* Number of molecules of DPPC, P, N, 2T-P, or 2T-N, divided evenly between the two bilayer leaflets. *^b^* “Plateau value” of the deuterium order parameter, <S>, where S = |½<3*cos*^2^*θ* - 1>|, in the highest order lipid tail region, i.e., averaged over carbons 4-8 85. Standard deviation ~0.006. *^c^* Area per lipid—counting prodrugs (but not parent compounds) in the “lipid” total. Standard deviation for simulations ~2 Å^2^. *^d^* Time-averaged number of ion-ion and ion-dipole interactions among headgroups and prodrug linker moieties, not distinguished. Percentage of total possible contacts given in parentheses. *^e^* Order parameter plateau value 86; area per lipid 87.

**Table 2:** Cell viability data of HeLa (cervical cancer), MCF7 (breast cancer), MCF10A (normal breast) cell lines after 48h treatment with free parent compounds or prodrug incorporated liposomes, n=4.

Cell Lines	P [μM]	2T-P [μM]	N [μM]	2T-N [μM]
HeLa	0.009 ± 0.001	1.373 ± 0.044	0.005 ± 0.001	0.020 ± 0.002
MCF7	0.009 ± 0.004	2.556 ± 0.126	0.003 ± 0.000	0.038 ± 0.007
MCF10A	0.014 ± 0.004	1.537 ± 0.130	0.005 ± 0.000	1.105 ± 0.131

## Abbreviations

CH_2_Cl_2_: methylene chloride
DCC: N,N-dicyclohexyl-carbodiimide
DMAP: 3- aminopyrazole, 4-dimethylaminopyridine
DMF: dimethylformamide
DMSO: 5- bromovanillin, dimethyl sulfoxide
DLS: dynamic light scatter
DPPA: 1,2 - dipalmitoyl -sn- glycero -3- phophate (monosodium salt)
DPPC: 1,2- dipalmitoly-sn-glycero-3-phosphocholine
DPPE-Glu: N-Glutaryl-L-α-phosphatidylethanolamine, Dipalmitoyl
DSC: differential scanning calorimetry
DSPE-PEG2000: 1,2-distearoyl-sn-glycerol-3-phosphoethanolamin-N-[methoxy (polyethylene glycol) -2000] ammonium salt
DSPE-PEG5000: 1,2 - distearoyl -sn- glycero -3- phospho- ethano lamine -N- (polyethylene glycol) -5000) (ammonium salt)
EtOH: ethanol
MB: microbubble
MeOH: methanol
MTT: 3-(4,5-dimethylthiazol-2-yl)-2,5-diphenyl tetrazolium bromide
N: 7-(3,5-Dibromophenyl)-2-hydroxy-7,11-dihydrobenzo[h]-furo[3,4-b]quinolin-8(10H)-one
NMR: nuclear magnetic resonance
P: podophyllotoxin
PBS: phosphate buffered saline
PLL: prodrug-loaded liposomes
TLC: thin layer chromatography
2T-P: sodium 2,3-bis (palmitoyloxy) propyl (2-(5-oxo-5- (((5S,5aS,8aS,9S)-8-oxo-9-(3,4,5-trimethoxyphenyl)-5,5a,6,8,8a,9-hexahydrofuro[3',4':6,7]naphtha[2,3-d][1,3]dioxol-5-yl)oxy) pentanamido) ethyl) phosphate
2T-N: sodium 2,3-bis(palmitoyloxy) propyl (2-(5-((7-(3,5-dibromophenyl)-8-oxo-7,8,10,11-tetrahydrobenzo[h]furo[3,4-b]quinolin-2-yl) oxy) -5-oxopentanamido) ethyl) phosphate

## References

[1] Neoptolemos JP, Kleeff J, Michl P, Costello E, Greenhalf W, Palmer DH (2018). Therapeutic developments in pancreatic cancer: current and future perspectives. Nat Rev Gastroenterol Hepatol.

[2] Bockhorn M, Uzunoglu FG, Adham M, Imrie C, Milicevic M, Sandberg AA (2014). Borderline resectable pancreatic cancer: a consensus statement by the International Study Group of Pancreatic Surgery (ISGPS). Surgery.

[3] Tartis MS, Kruse DE, Zheng H, Zhang H, Kheirolomoom A, Marik J (2008). Dynamic microPET imaging of ultrasound contrast agents and lipid delivery. J Control Release.

[4] Andresen TL, Jensen SS, Jorgensen K (2005). Advanced strategies in liposomal cancer therapy: problems and prospects of active and tumor specific drug release. Prog Lipid Res.

[5] Bhattacharya S, Bajaj A (2007). Membrane-forming properties of gemini lipids possessing aromatic backbone between the hydrocarbon chains and the cationic headgroup. J Phys Chem B.

[6] Borden MA, Martinez GV, Ricker J, Tsvetkova N, Longo M, Gillies RJ (2006). Lateral phase separation in lipid-coated microbubbles. Langmuir.

[7] Sirsi S, Borden M (2009). Microbubble Compositions, Properties and Biomedical Applications. Bubble Sci Eng Technol.

[8] Sheeran PS, Luois S, Dayton PA, Matsunaga TO (2011). Formulation and acoustic studies of a new phase-shift agent for diagnostic and therapeutic ultrasound. Langmuir.

[9] Bildstein L, Dubernet C, Couvreur P (2011). Prodrug-based intracellular delivery of anticancer agents. Adv Drug Deliv Rev.

[10] Irby D, Du C, Li F (2017). Lipid-Drug Conjugate for Enhancing Drug Delivery. Mol Pharm.

[11] Golan T, Grenader T, Ohana P, Amitay Y, Shmeeda H, La-Beck NM (2015). Pegylated liposomal mitomycin C prodrug enhances tolerance of mitomycin C: a phase 1 study in advanced solid tumor patients. Cancer Med.

[12] Ferrara K, Pollard R, Borden M (2007). Ultrasound microbubble contrast agents: fundamentals and application to gene and drug delivery. Annu Rev Biomed Eng.

[13] Song K-H, Trudeau T, Kar A, Borden MA, Gutierrez-Hartmann A (2019). Ultrasound-mediated delivery of siESE complexed with microbubbles attenuates HER2+/- cell line proliferation and tumor growth in rodent models of breast cancer. Nanotheranostics.

[14] Dubinsky TJ, Cuevas C, Dighe MK, Kolokythas O, Hwang JH (2008). High-intensity focused ultrasound: current potential and oncologic applications. AJR Am J Roentgenol.

[15] He SX XL, Yao SS, Yu JS, Lan J, Yu QH (1999). The preclinical research of high intensity focused ultrasound. Beijing Yikedaxue Xue Bao.

[16] D'Souza JC, Sultan LR, Hunt SJ, Gade TP, Karmacharya MB, Schultz SM (2019). Microbubble-enhanced ultrasound for the antivascular treatment and monitoring of hepatocellular carcinoma. Nanotheranostics.

[17] Li T, Wang YN, Khokhlova TD, D'Andrea S, Starr F, Chen H (2015). Pulsed High-Intensity Focused Ultrasound Enhances Delivery of Doxorubicin in a Preclinical Model of Pancreatic Cancer. Cancer Res.

[18] Pitt WG, Husseini GA, Staples BJ (2004). Ultrasonic drug delivery-a general review. Expert Opin Drug Deliv.

[19] Tartis MS, McCallan J, Lum AF, LaBell R, Stieger SM, Matsunaga TO (2006). Therapeutic effects of paclitaxel-containing ultrasound contrast agents. Ultrasound Med Biol.

[20] Xiong F, Nirupama S, Sirsi SR, Lacko A, Hoyt K (2017). Ultrasound-Stimulated Drug Delivery Using Therapeutic Reconstituted High-Density Lipoprotein Nanoparticles. Nanotheranostics.

[21] Lamb YN, Scott LJ (2017). Liposomal Irinotecan: A Review in Metastatic Pancreatic Adenocarcinoma. Drugs.

[22] Bulbake UDS, Kommineni N, Khan W (2017). Liposomal Formulations in Clinical Use: An Updated Review.

[23] Nguyen AT, Lewin PA, Wrenn SP (2015). Hydrophobic drug concentration affects the acoustic susceptibility of liposomes. Biochim Biophys Acta.

[24] Khan DR, Rezler EM, Lauer-Fields J, Fields GB (2008). Effects of drug hydrophobicity on liposomal stability. Chem Biol Drug Des.

[25] Yu G, Yang Z, Fu X, Yung BC, Yang J, Mao Z (2018). Polyrotaxane-based supramolecular theranostics. Nat Commun.

[26] Zaro JL (2015). Lipid-based drug carriers for prodrugs to enhance drug delivery. AAPS J.

[27] Klibanov AL, Shevchenko TI, Raju BI, Seip R, Chin CT (2010). Ultrasound-triggered release of materials entrapped in microbubble-liposome constructs: A tool for targeted drug delivery. Journal of Controlled Release.

[28] Kheirolomoom A, Dayton PA, Lum AF, Little E, Paoli EE, Zheng H (2007). Acoustically-active microbubbles conjugated to liposomes: characterization of a proposed drug delivery vehicle. J Control Release.

[29] Geers B, Lentacker I, Sanders NN, Demeester J, Meairs S, De Smedt SC (2011). Self-assembled liposome-loaded microbubbles: The missing link for safe and efficient ultrasound triggered drug-delivery. Journal of Controlled Release.

[30] Cool SK, Geers B, Roels S, Stremersch S, Vanderperren K, Saunders JH (2013). Coupling of drug containing liposomes to microbubbles improves ultrasound triggered drug delivery in mice. Journal of Controlled Release.

[31] Jing Liu DZ (2016). Highly enhanced leukemia therapy and oral bioavailability from a novel amphiphilic prodrug of cytarabine. RSC Advantages.

[32] Senter PD, Schreiber GJ, Hirschberg DL, Ashe SA, Hellstrom KE, Hellstrom I (1989). Enhancement of the in vitro and in vivo antitumor activities of phosphorylated mitomycin C and etoposide derivatives by monoclonal antibody-alkaline phosphatase conjugates. Cancer Res.

[33] Wang Y, Wang X, Deng F, Zheng N, Liang Y, Zhang H (2018). The effect of linkers on the self-assembling and anti-tumor efficacy of disulfide-linked doxorubicin drug-drug conjugate nanoparticles. J Control Release.

[34] Magedov IV, Frolova L, Manpadi M, Bhoga U, Tang H, Evdokimov NM (2011). Anticancer properties of an important drug lead podophyllotoxin can be efficiently mimicked by diverse heterocyclic scaffolds accessible via one-step synthesis. J Med Chem.

[35] Mura S, Bui DT, Couvreur P, Nicolas J (2015). Lipid prodrug nanocarriers in cancer therapy. J Control Release.

[36] Rautio J, Kumpulainen H, Heimbach T, Oliyai R, Oh D, Jarvinen T (2008). Prodrugs: design and clinical applications. Nat Rev Drug Discov.

[37] Fang S, Niu Y, Zhu W, Zhang Y, Yu L, Li X (2015). Liposomes assembled from a dual drug-tailed phospholipid for cancer therapy. Chem Asian J.

[38] Johnston RK, Harper JC, Tartis MS (2017). Control over Silica Particle Growth and Particle-Biomolecule Interactions Facilitates Silica Encapsulation of Mammalian Cells with Thickness Control. ACS Biomater Sci Eng.

[39] Ferrara K, Dayton P, Shortencarrier M, Kruse D (2003). Ultrasound contrast agents and their use in monitoring therapy.

[40] Shortencarier MJ, Dayton PA, Bloch SH, Schumann PA, Matsunaga TO, Ferrara KW (2004). A method for radiation-force localized drug delivery using gas-filled lipospheres. IEEE Transactions on Ultrasonics, Ferroelectrics, and Frequency Control.

[41] Dickson CJ, Madej BD, Skjevik AA, Betz RM, Teigen K, Gould IR (2014). Lipid14: The Amber Lipid Force Field. J Chem Theory Comput.

[42] Wang J, Wolf RM, Caldwell JW, Kollman PA, Case DA (2004). Development and testing of a general Amber force field. Journal of Computational Chemistry.

[43] Bayly CI, Cieplak P, Cornell W, Kollman PA (1993). A well-behaved electrostatic potential based method using charge restraints for deriving atomic ….

[44] Dupradeau F-Y, Pigache A, Zaffran T, Savineau C, Lelong R, Grivel N (2010). The R.E.D. tools: advances in RESP and ESP charge derivation and force field library building. Physical chemistry chemical physics: PCCP.

[45] Frisch MJ, Trucks GW, Schlegel HB, Scuseria GE, Robb MA, Cheeseman JR (2009). Gaussian 09. Wallingford, CT: Gaussian, Inc.

[46] Skjevik AA, Madej BD, Walker RC, Teigen K (2012). LIPID11: A modular framework for lipid simulations using Amber. Journal of Physical Chemistry B.

[47] Case DA, Berryman JT, Betz RM, Cerutti DS, Cheatham TE, III, Darden TA (2015). AMBER 2015.

[48] Jo S, Lim JB, Klauda JB, Im W (2009). CHARMM-GUI membrane builder for mixed bilayers and its application to yeast membranes. Biophysical Journal.

[49] Jorgensen WL, Jenson C (1998). Temperature dependence of TIP3P, SPC, and TIP4P water from NPT Monte Carlo simulations: Seeking temperatures of maximum density. Journal of Computational Chemistry.

[50] Salomon-Ferrer R, Götz AW, Poole D, Le Grand S, Walker RC (2013). Routine microsecond molecular dynamics simulations with AMBER on GPUs. 2. Explicit solvent particle mesh Ewald. Journal of Chemical Theory and Computation.

[51] Berendsen HJC, Postma JPM, van Gunsteren WF, DiNola A, Haak JR (1984). Molecular dynamics with coupling to an external bath. The Journal of Chemical Physics.

[52] Darden T, York D, Pedersen L (1993). Particle mesh Ewald: An N∙log(N) method for Ewald sums in large systems. The Journal of Chemical Physics.

[53] Roe DR, Cheatham TE (2013). PTRAJ and CPPTRAJ: Software for processing and analysis of molecular dynamics trajectory data. Journal of Chemical Theory and Computation.

[54] Schrödinger LLC The PyMOL Molecular Graphics System, Version 1.7.6.5. Schrödinger LLC.

[55] Escobar JFB, Restrepo MHP, Fernandez DMM, Martinez AM, Giordani C, Castelli F (2018). Synthesis and interaction of sterol-uridine conjugate with DMPC liposomes studied by differential scanning calorimetry. Colloids Surf B Biointerfaces.

[56] Demetzos C (2008). Differential Scanning Calorimetry (DSC): a tool to study the thermal behavior of lipid bilayers and liposomal stability. J Liposome Res.

[57] Wei X, Patil Y, Ohana P, Amitay Y, Shmeeda H, Gabizon A (2017). Characterization of Pegylated Liposomal Mitomycin C Lipid-Based Prodrug (Promitil) by High Sensitivity Differential Scanning Calorimetry and Cryogenic Transmission Electron Microscopy. Mol Pharm.

[58] Mikhalin AA (2015). Lipophilic prodrug conjugates allow facile and rapid synthesis of high-loading capacity liposomes without the need for post-assembly purification. Journal of Liposome Research.

[59] Torchilin V (2003). Liposomes. A practical approach.: Oxford: Oxford University Press.

[60] Pedersen TB (2002). A calorimetric study of phosphocholine membranes mixed with desmopressin and its diacylated prodrug derivative (DPP).

[61] Gulati M, Grover M, Singh S, Singh M (1998). Lipophilic drug derivatives in liposomes. Int J Pharmaceut.

[62] Hong SS, Kim SH, Lim SJ (2015). Effects of triglycerides on the hydrophobic drug loading capacity of saturated phosphatidylcholine-based liposomes. Int J Pharm.

[63] Giuseppina Bozzuto AM (2015). Liposomes as nanomedical devices.

[64] Alekseeva AA, Moiseeva EV, Onishchenko NR, Boldyrev IA, Singin AS, Budko AP (2017). Liposomal formulation of a methotrexate lipophilic prodrug: assessment in tumor cells and mouse T-cell leukemic lymphoma. Int J Nanomedicine.

[65] Abolmaali SS, Tamaddon AM, Dinarvand R (2013). A review of therapeutic challenges and achievements of methotrexate delivery systems for treatment of cancer and rheumatoid arthritis. Cancer Chemother Pharmacol.

[66] Kumar AA, Freisheim JH, Kempton RJ, Anstead GM, Black AM, Judge L (1983). Synthesis and characterization of a fluorescent analogue of methotrexate. J Med Chem.

[67] Cao Y, Chen Y, Yu T, Guo Y, Liu F, Yao Y (2018). Drug Release from Phase-Changeable Nanodroplets Triggered by Low-Intensity Focused Ultrasound. Theranostics.

[68] Meel Rvd (2014). Extracellular vesicles as drug delivery systems: Lessons from the liposome field.

[69] Surianarayanan R (2016). Effect of sample Concentration on the Characterization of Liposomes using Dynamic light Scattering Technique. Pharmaceutical Methods.

[70] Gro Smistad SB, Siv Jorunn Alund, Anne Berit C (2012). Samuelsen, Marianne Hiorth. The potential of pectin as a stabilizer for liposomal drug delivery systems, Carbohydrate Polymers.

[71] Coimbra M, Isacchi B, van Bloois L, Torano JS, Ket A, Wu X (2011). Improving solubility and chemical stability of natural compounds for medicinal use by incorporation into liposomes. Int J Pharm.

[72] Godefridus J (2011). Peters ADA, Irene V. Bijnsdorp & Marit L. Sandvold. Lipophilic Prodrugs and Formulations of Conventional (Deoxy)Nucleoside and Fluoropyrimidine Analogs in Cancer. Nucleosides, Nucleotides and Nucleic Acids.

[73] Barenholz Y, Amselem S, Goren D, Cohen R, Gelvan D, Samuni A (1993). Stability of liposomal doxorubicin formulations: problems and prospects. Med Res Rev.

[74] Soundararajan A, Bao A, Phillips WT, Perez R 3rd, Goins BA (2009). [(186)Re]Liposomal doxorubicin (Doxil): in vitro stability, pharmacokinetics, imaging and biodistribution in a head and neck squamous cell carcinoma xenograft model. Nucl Med Biol.

[75] Kyrychenko A, Ladokhin AS (2013). Molecular dynamics simulations of depth distribution of spin-labeled phospholipids within lipid bilayer. Journal of Physical Chemistry B.

[76] Angles G, Dotson R, Bueche K, Pias SC (2017). Predicted Decrease in Membrane Oxygen Permeability with Addition of Cholesterol. Adv Exp Med Biol.

[77] Fouladi F, Steffen KJ, Mallik S (2017). Enzyme-Responsive Liposomes for the Delivery of Anticancer Drugs. Bioconjug Chem.

[78] Diener C, Muñoz-Gonzalez F, Encarnación S, Resendis-Antonio O (2016). The space of enzyme regulation in HeLa cells can be inferred from its intracellular metabolome. Scientific Reports.

[79] Dong H, Pang L, Cong H, Shen Y, Yu B (2019). Application and design of esterase-responsive nanoparticles for cancer therapy. Drug delivery.

[80] Wang WQ, Liu L, Xu JZ, Yu XJ (2019). Reflections on depletion of tumor stroma in pancreatic cancer. Biochim Biophys Acta Rev Cancer.

[81] Kim EJ, Sahai V, Abel EV, Griffith KA, Greenson JK, Takebe N (2014). Pilot clinical trial of hedgehog pathway inhibitor GDC-0449 (vismodegib) in combination with gemcitabine in patients with metastatic pancreatic adenocarcinoma. Clin Cancer Res.

[82] Liu Y, Tamam H, Yeo Y (2018). Mixed Liposome Approach for Ratiometric and Sequential Delivery of Paclitaxel and Gemcitabine. AAPS PharmSciTech.

[83] Meng J (2016). Combination Therapy using Co-encapsulated Resveratrol and Paclitaxel in Liposomes for Drug Resistance Reversal in Breast Cancer Cells in vivo. Scientific Reports.

[84] Lavi O, Gottesman MM, Levy D (2012). The dynamics of drug resistance: a mathematical perspective. Drug Resist Updat.

[85] Venable RM, Brown FLH, Pastor RW (2015). Mechanical properties of lipid bilayers from molecular dynamics simulation. Chemistry and Physics of Lipids.

[86] Petrache HI, Dodd SW, Brown MF (2000). Area per lipid and acyl length distributions in fluid phosphatidylcholines determined by <sup>2</sup>H NMR spectroscopy. Biophys J.

[87] Kučerka N, Nieh M-P, Katsaras J (2011). Fluid phase lipid areas and bilayer thicknesses of commonly used phosphatidylcholines as a function of temperature. Biochimica et Biophysica Acta (BBA) - Biomembranes.

