# Supplementary material for: Phospholipid prodrug conjugates of insoluble chemotherapeutic agents for ultrasound targeted drug delivery
Source: Nanotheranostics. 2020 Jan 1;4(1):40–56. doi: 10.7150/ntno.37738 (PMC6940203; doi:10.7150/ntno.37738)
Supplement: Supplementary file 1 — Supplementary figures and tables. [file ntnov04p0040s1.pdf]

Supplemental Methods and Data

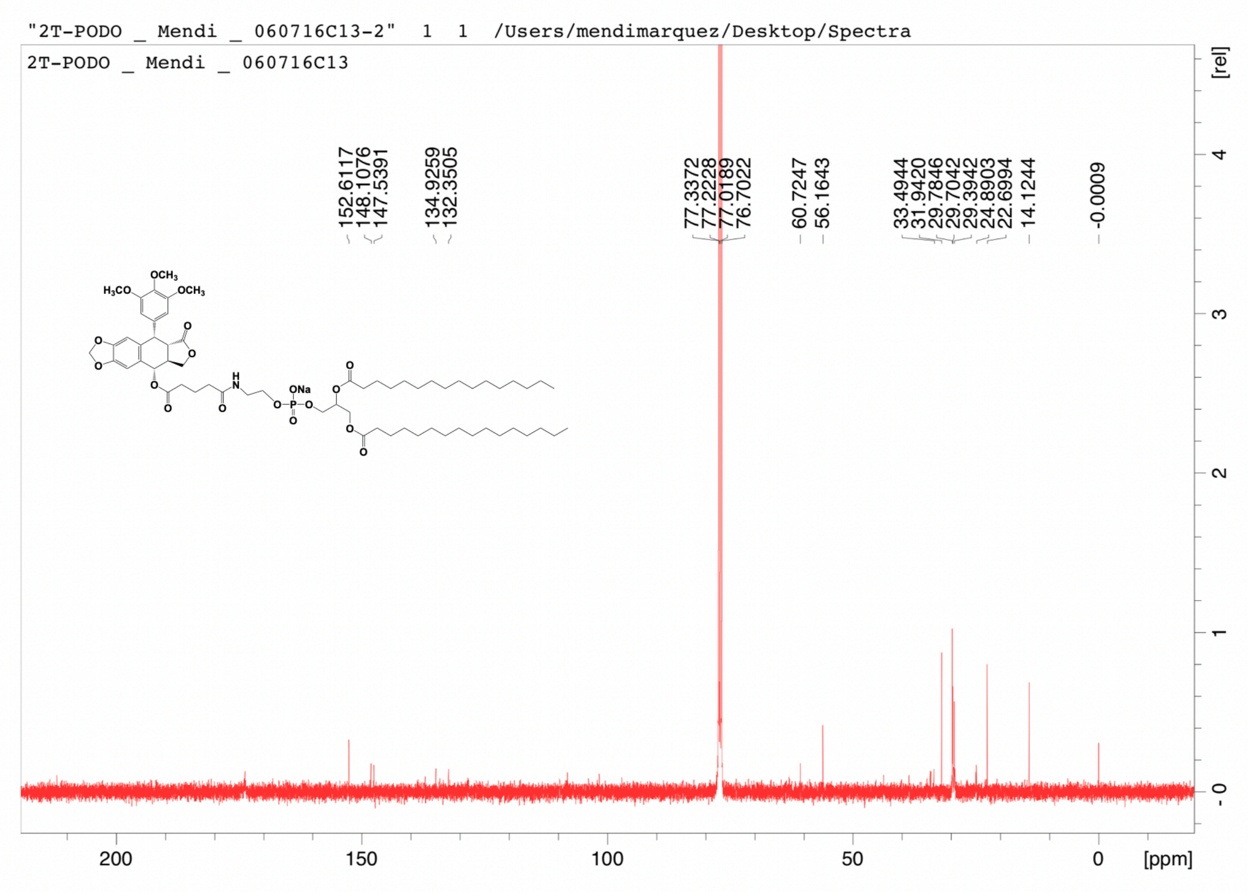

Figure S1: ^{13}C NMR spectrum of 2T-P in $CDCl_3$.

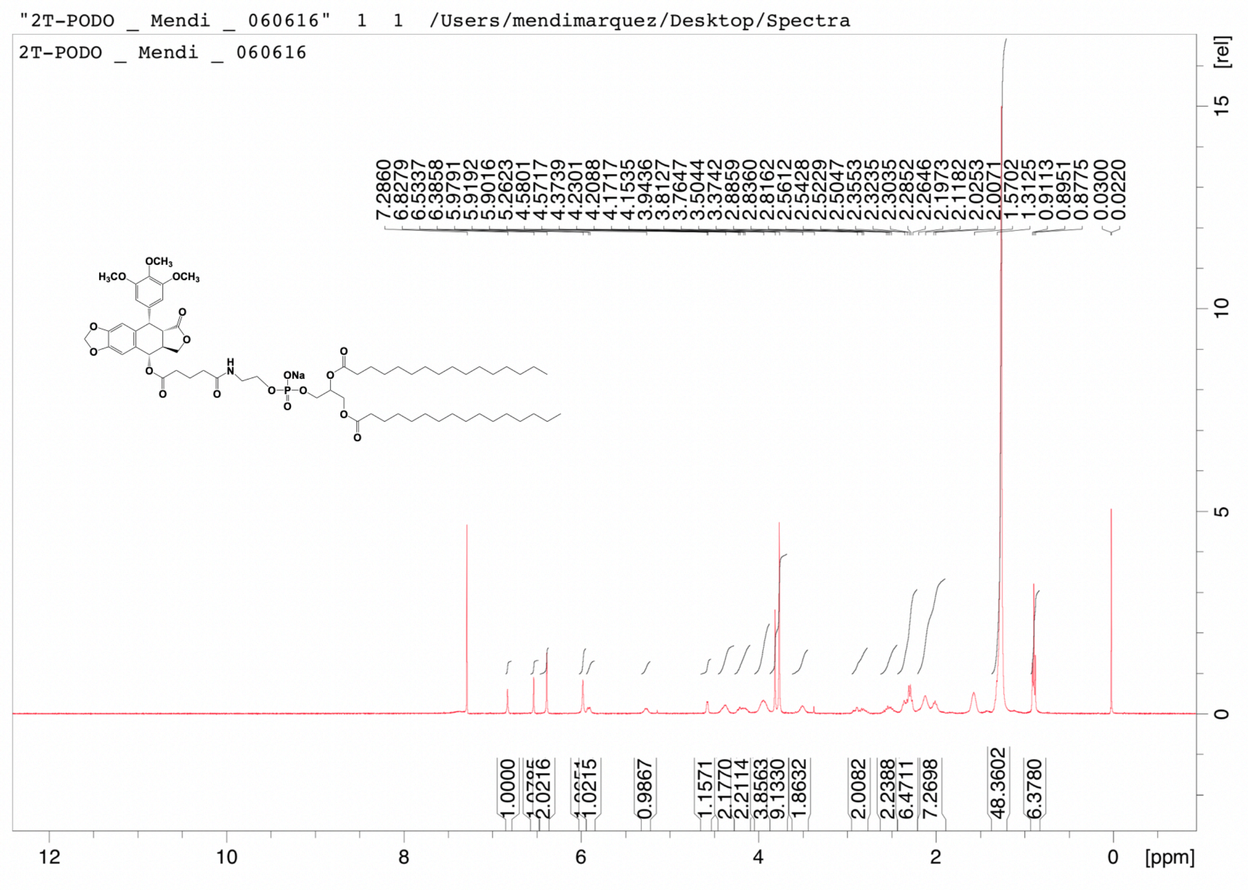

Figure S2: ^{1}H NMR spectrum of 2T-P in $CDCl_3$.

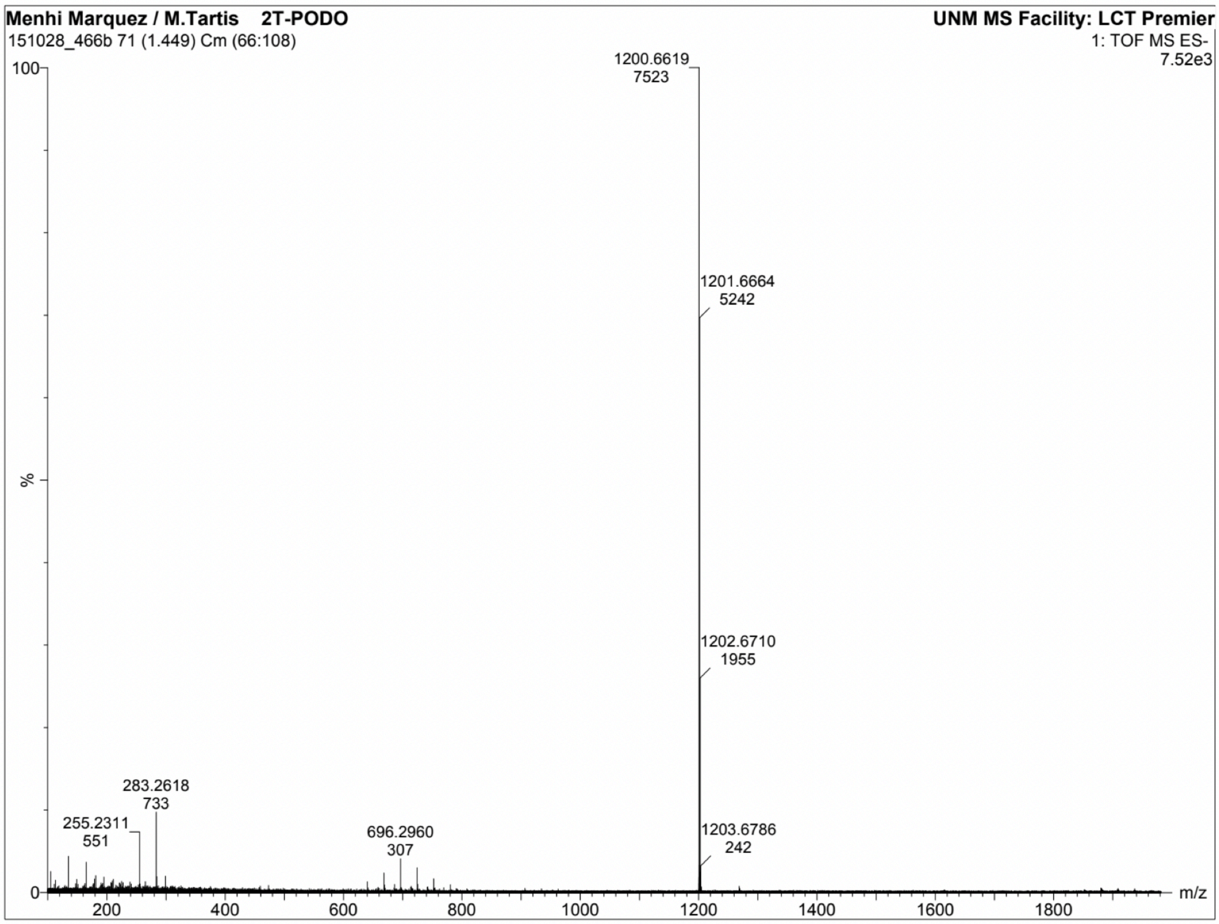

Figure S3: Mass spectrometry elemental composition spectra.

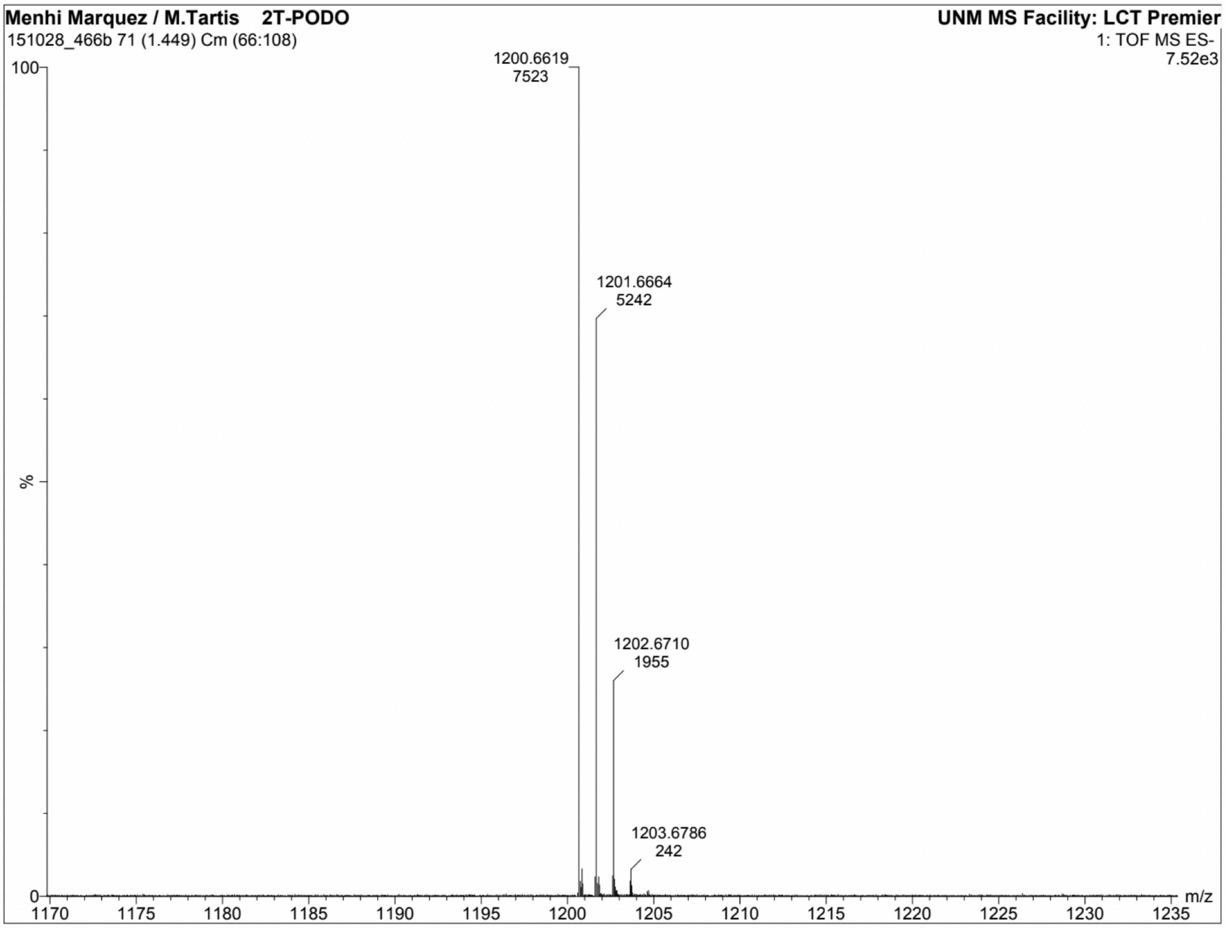

Figure S4: Mass spectrometry elemental composition spectra continued.

Elemental Composition Report

Single Mass Analysis

Tolerance = 20.0 PPM / DBE: min = -5.0, max = 75.0
Isotope cluster parameters: Separation = 1.0 Abundance = 1.0%

Monoisotopic Mass, Odd and Even Electron Ions
449 formula(e) evaluated with 7 results within limits (up to 50 closest results for each mass)

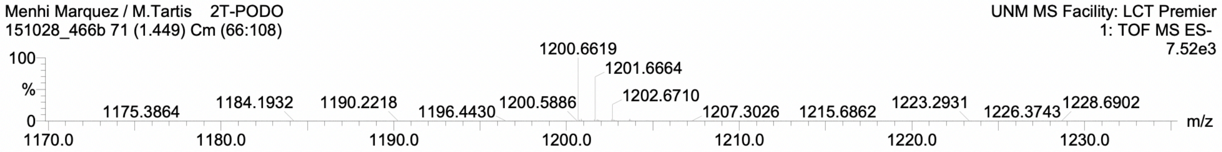

Minimum:				-5.0		
Maximum:		200.0	20.0	75.0		
Mass	Calc. Mass	mDa	PPM	DBE	Score	Formula
1200.6619	1200.6600	1.9	1.6	16.5	2	C64 H99 N O18 P
	1200.6576	4.3	3.6	13.5	1	C62 H100 N O18 Na P
	1200.6514	10.5	8.7	21.0	5	C68 H97 O16 P
	1200.6490	12.9	10.7	18.0	3	C66 H98 O16 Na P
	1200.6422	19.7	16.4	16.5	7	C64 H99 N O16 P 32S
	1200.6398	22.1	18.4	13.5	6	C62 H100 N O16 Na P 32S
	1200.6389	23.0	19.2	21.5	4	C67 H95 N O16 P

Figure S5: Mass spectrometry elemental composition report.

Figures S1-S5: 2T-P Spectral Descriptions

sodium 2,3-bis (palmitoyloxy) propyl (2-(5-oxo-5- (((5S,5aS,8aS,9S)-8- oxo-9- (3,4,5-trimethoxyphenyl)- 5,5a,6,8,8a,9- hexahydrofuro [3',4':6,7] naphtha [2,3-d][1,3]dioxol-5-yl) oxy) pentanamido) ethyl) phosphate (2T-P). 28.6% yield as white solid, mp = 60°C (CH_2Cl_2/MeOH = 7/1). ^{1}H NMR ($CDCl_3$– d_6) 6.83 (s, 1H), 6.53 (s, 1H), 6.39 (s, 2H), 5.98 (s, 2H), 5.91 (d, J=7.04 Hz, 1H), 5.26 (s, 1H) , 4.58 (d, J=3.32, 1H), 4.37 (s, 2H), 4.23-4.15 (m, 2H), 3.94 (s, 4H), 3.79 (d, J=19.2 Hz, 9H), 3.50 (s, 2H), 2.89-2.82 (m, 2H), 2.56-2.50 (m, 2H), 2.35-2.19 (m, 6H), 2.12-2.01 (m, 7H), 1.31 (s, 48H), 0.91-0.88 (m, 6H); ^{13}C NMR ($CDCl_3$– d_6) 173.8, 152.6, 148.1, 147.5, 134.9, 132.4, 108.2, 101.7, 60.7, 56.2, 38.7, 34.5, 34.3, 33.5, 31.9, 29.8, 29.7, 29.4, 25, 24.9, 22.7, 14.1

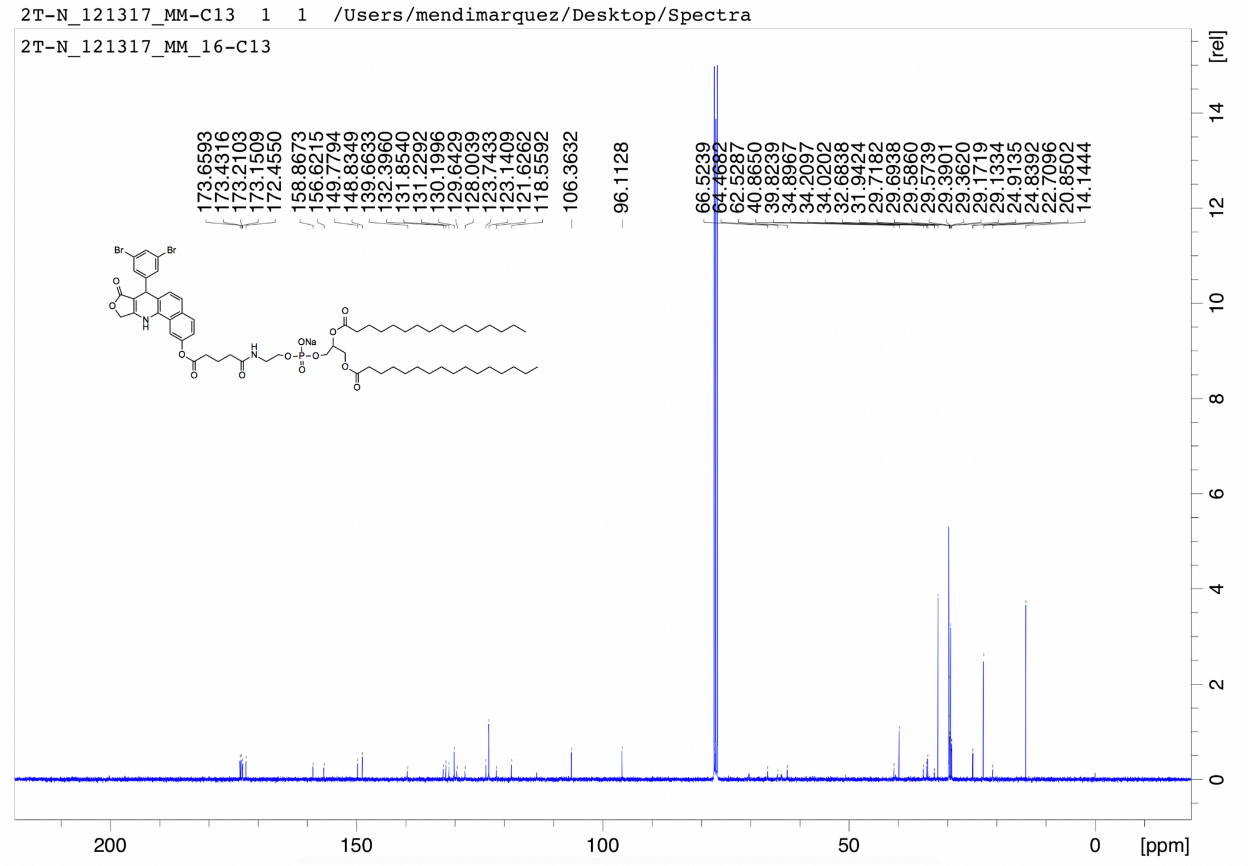

Figure S6: ^{13}C NMR spectrum of 2T-N in $CDCl_3$.

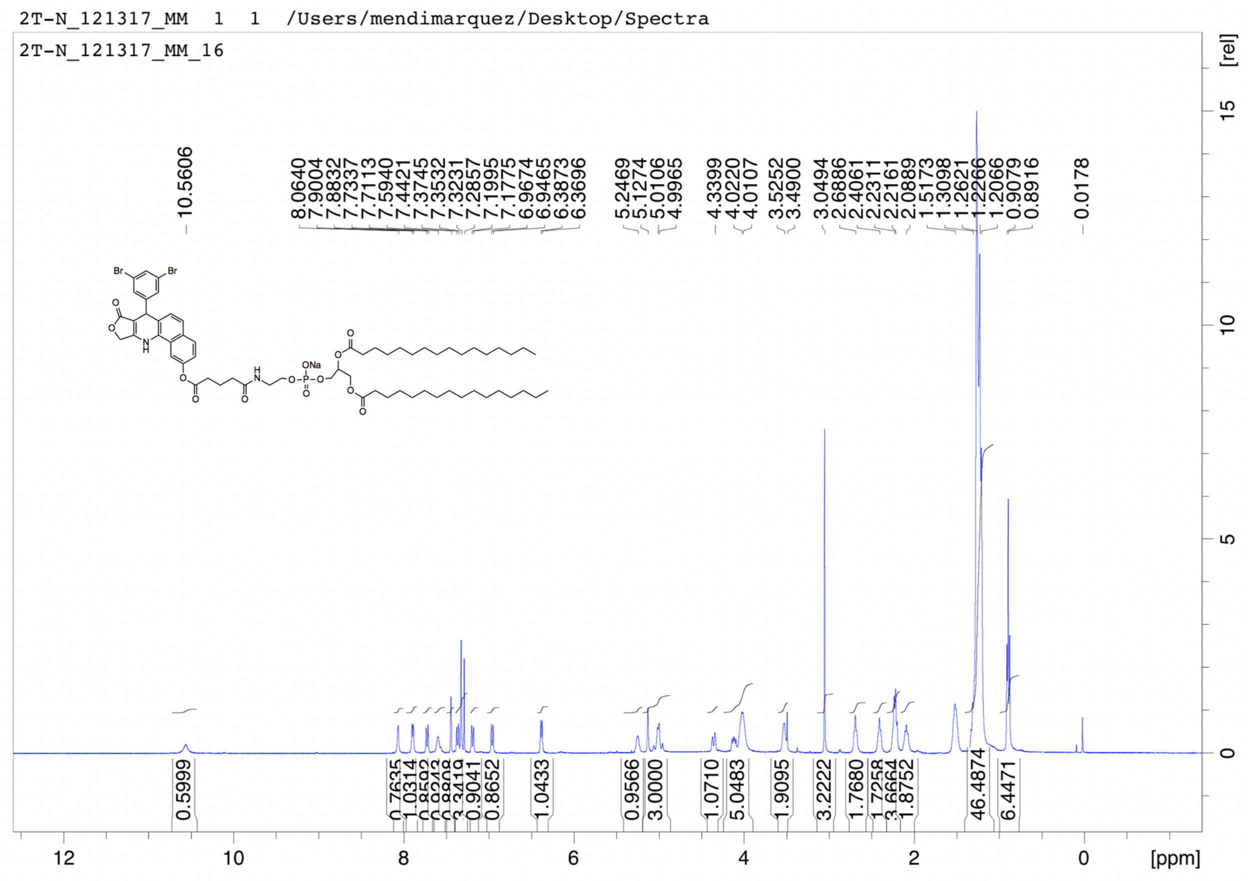

Figure S7: ^{1}H NMR spectrum of 2T-N in $CDCl_3$.

Figures S6-S7: 2T-N Spectral Descriptions

sodium 2,3-bis(palmitoyloxy) propyl (2-(5-((7-(3,5 -dibromophenyl) -8-oxo-7,8,10,11-tetrahydrobenzo [h]furo[3,4-b]quinolin-2-yl) oxy)-5-oxopentanamido) ethyl) phosphate (2T-N). 25.6% yield as oil, (CH_2Cl_2/MeOH = 7/1). ^{1}H NMR ($CDCl_3$– d_6) 10.56 (s, 1H), 8.06 (s, 1H), 7.89 (d, *J*=6.88 Hz, 1H); 7.72 (d, *J*=8.96 Hz, 1H), 7.59 (s, 1H), 7.44 (s, 1H), 7.37-7.29 (m, 3H), 7.19 (d, *J*=8.8 Hz, 1H), 6.96 (d, *J*=8.36, 1H), 6.38 (d, *J*=7.08 Hz, 1H), 5.25 (s, 1H), 5.13-4.99 (m, 3H), 4.34 (d, *J*= , 1H), 4.02-4.01 (m, 5H), 3.51 (d, *J*=14.08 Hz, 2H), 3.05 (s, 3H), 2.69 (s, 2H), 2.41 (s, 2H), 2.23 (d, *J*=6Hz, 4H), 2.09 (s, 2H), 1.31-1.21 (m, 46H) 0.91-0.89 (m, 6H)**;** ^{13}C NMR ($CDCl_3$– d_6) 173.6, 173.4, 173.2, 173.2, 172.4 158.9, 156.6, 149.8, 148.8, 139.7, 132.4, 131.9, 131.2, 130.2, 129.6, 128.0, 123.7, 123.1, 121.6, 118.6, 106.4, 96.1, 66.5, 64.5, 62.5, 40.9, 39.8, 34.9, 34.2, 34.0, 32.7, 31.9, 29.7, 29.7, 29.6, 29.6, 29.4, 29.4, 29.2, 29.1, 24.9, 24.8, 22.7, 20.9, 14.1.

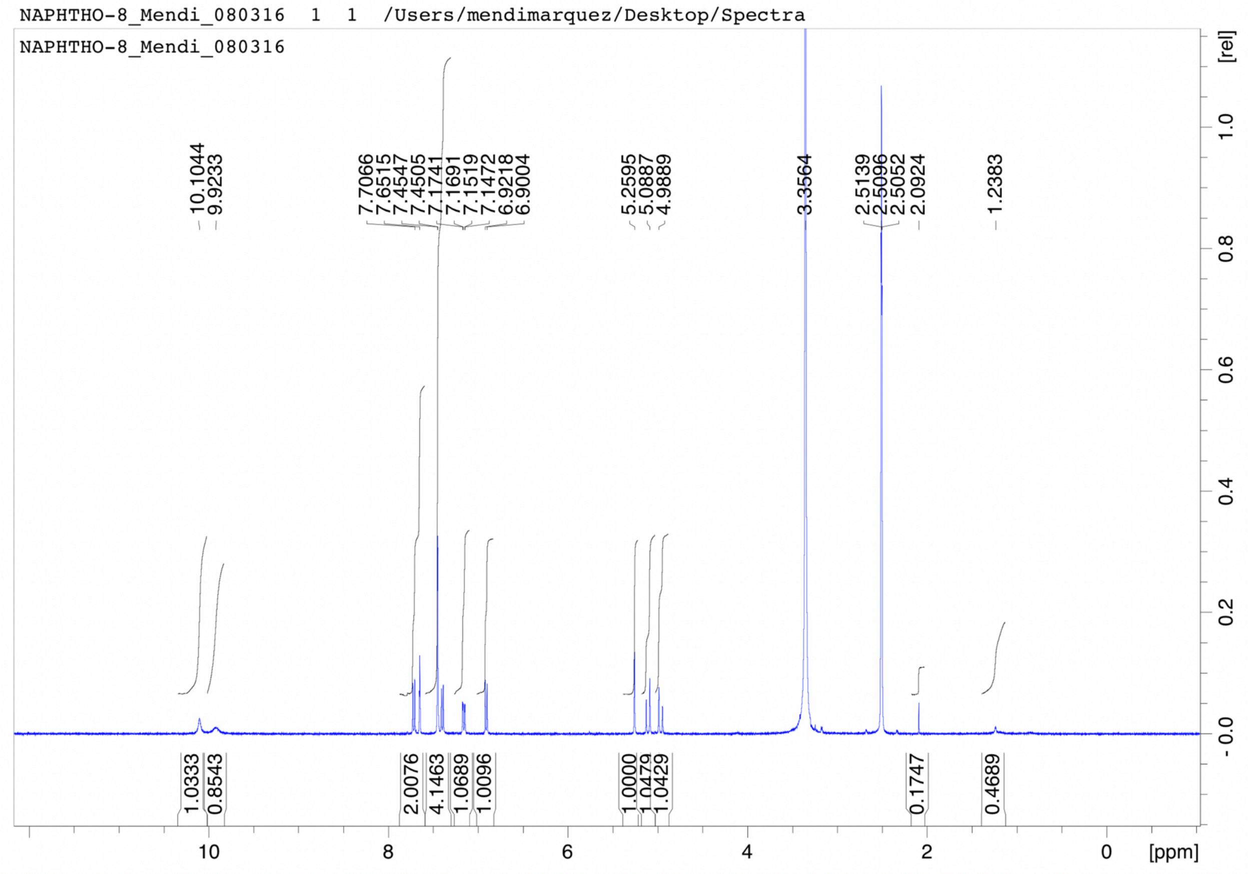

Figure S8: ^{1}H NMR spectrum of N in DMSO.

Figure S8: N Spectral Descriptions

7-(3,5-Dibromophenyl)-2-hydroxy-7,11-dihydrobenzo[h]- furo[3,4-b]quinolin-8(10H)-one (N). 50% yield as white solid, mp ≥ 290 ◦C ($CHCl_3$/MeOH = 20/1). ^{1}H NMR (DMSO-d_6) δ 4.92 5.12 (dd, J = 15 Hz, 2H), 5.24 (s, 1H), 6.88 (d, J = 9.0 Hz, 1H), 7.16 (d, J = 9.0 Hz, 1H), 7.37 7.71 (m, 6H), 9.90 (s, 1H), 10.09 (s, 1H).

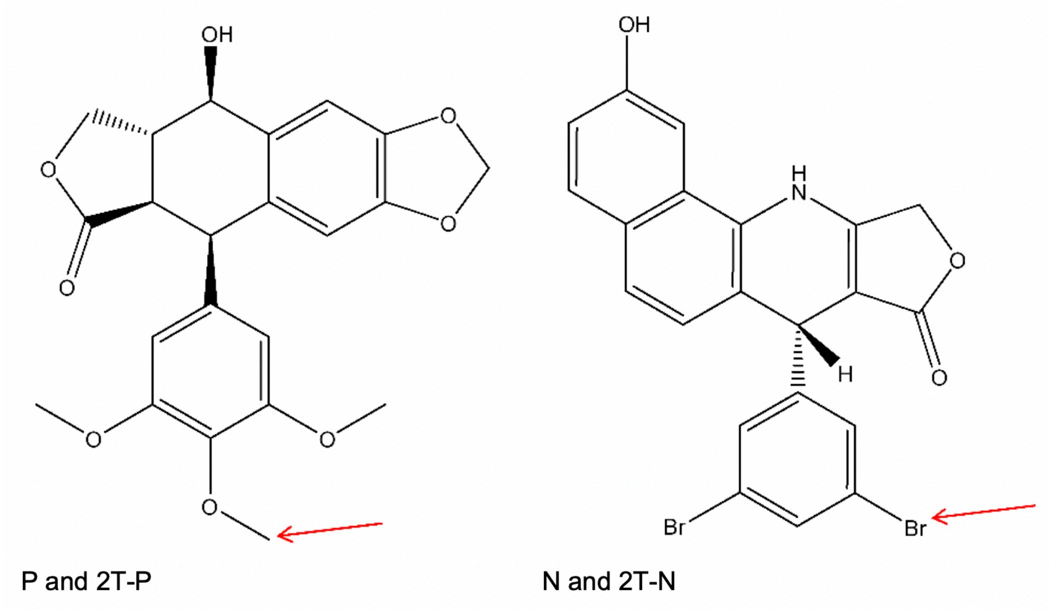

Figure S9: Arrows indicate the atoms used to track the compound positions in the molecular dynamic simulation results shown in Figure 8.

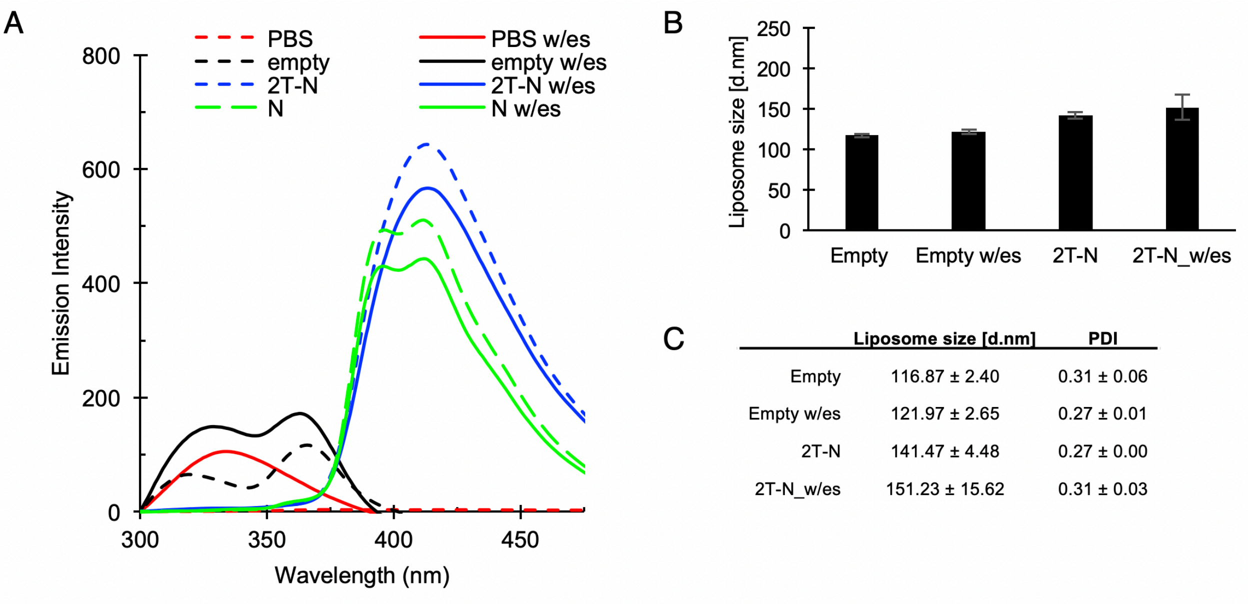

	Liposome size [d.nm]	PDI
Empty	116.87 ± 2.40	0.31 ± 0.06
Empty w/es	121.97 ± 2.65	0.27 ± 0.01
2T-N	141.47 ± 4.48	0.27 ± 0.00
2T-N_w/es	151.23 ± 15.62	0.31 ± 0.03

Figure S10: 2T-N degradation in the presence of porcine liver esterase.
(A) Spectrofluorophotometer representation of carboxylesterase assay, (B) average particle size distribution prior and post esterase, n=3, (C) tabulated particle size distribution, n=3. All samples are liposomes correlating to their respective lipid ratios: *empty* [DPPC: DSPE-PEG2000], *2T-N* [DPPC: DSPE-PEG2000: 2T-N], or *N* (parent compound) [DPPC: DSPE-PEG2000: N].

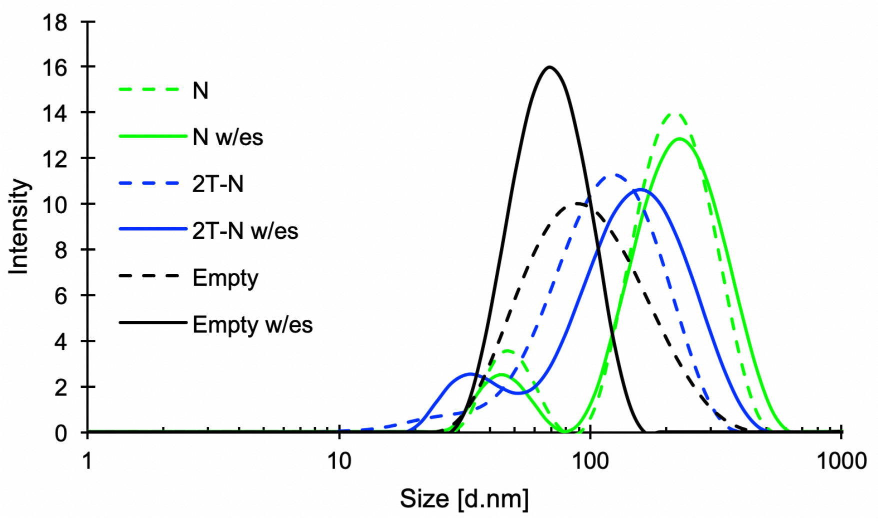

Figure S11: Representative size distribution of 2T-N and N loaded liposome degradation in the presence of porcine liver esterase. All samples are liposomes correlating to their respective lipid ratios: *empty* [DPPC: DSPE-PEG2000], *2T-N* [DPPC: DSPE-PEG2000: 2T-N], or *N* (parent compound) [DPPC: DSPE-PEG2000: N].

Figures S10 and S11:Esterase incubation validates N release from 2T-N

To demonstrate activation of the prodrugs, 2T-N loaded liposomes were incubated with porcine liver esterase and measured overtime using a spectrofluorophotometer to model it's susceptibility to hydrolysis at the carboxylic ester bond by carboxylesterases. 2T-P was not analyzed with this method due to it's lack of fluorescence. Esterase diluted in PBS gave an emission peak at ~334 nm, which it retained when added to empty liposomes (Figure 10). 2T-N liposomes had an emission peak at ~412 nm wherein the intensity decreased upon esterase incubation. This decrease in fluorescence intensity combined with the appearance of a subpopulation upon esterase incubation suggests the esterase altered 2T-N. Figure S10 B-C and S11, show a modest increase in size distribution and polydispersity in the presence of esterase compared with empty liposomes. P loaded liposomes sizes are not reported for the enzymatic assay due to the instability and bimodal size presence without additional purifying steps. No subpopulation is present within the empty liposomes before or after esterase incubation, however there is a subpopulation in the N loaded liposomes, which stands to reason that the subpopulation contains N cleaved from the 2T-loaded liposomes. Upon incorporation into lipid-based carriers, this activation method shows promise in drug resistance and potentially longer circulation by selective inhibition by the activating enzyme. Improving the pharmacokinetics and preventing premature drug leakage is essential in overcoming the current hurdles of toxicity in the clinic. This strategy could provide lipid-based carriers increased time to reach the targeted location and enter the cell.

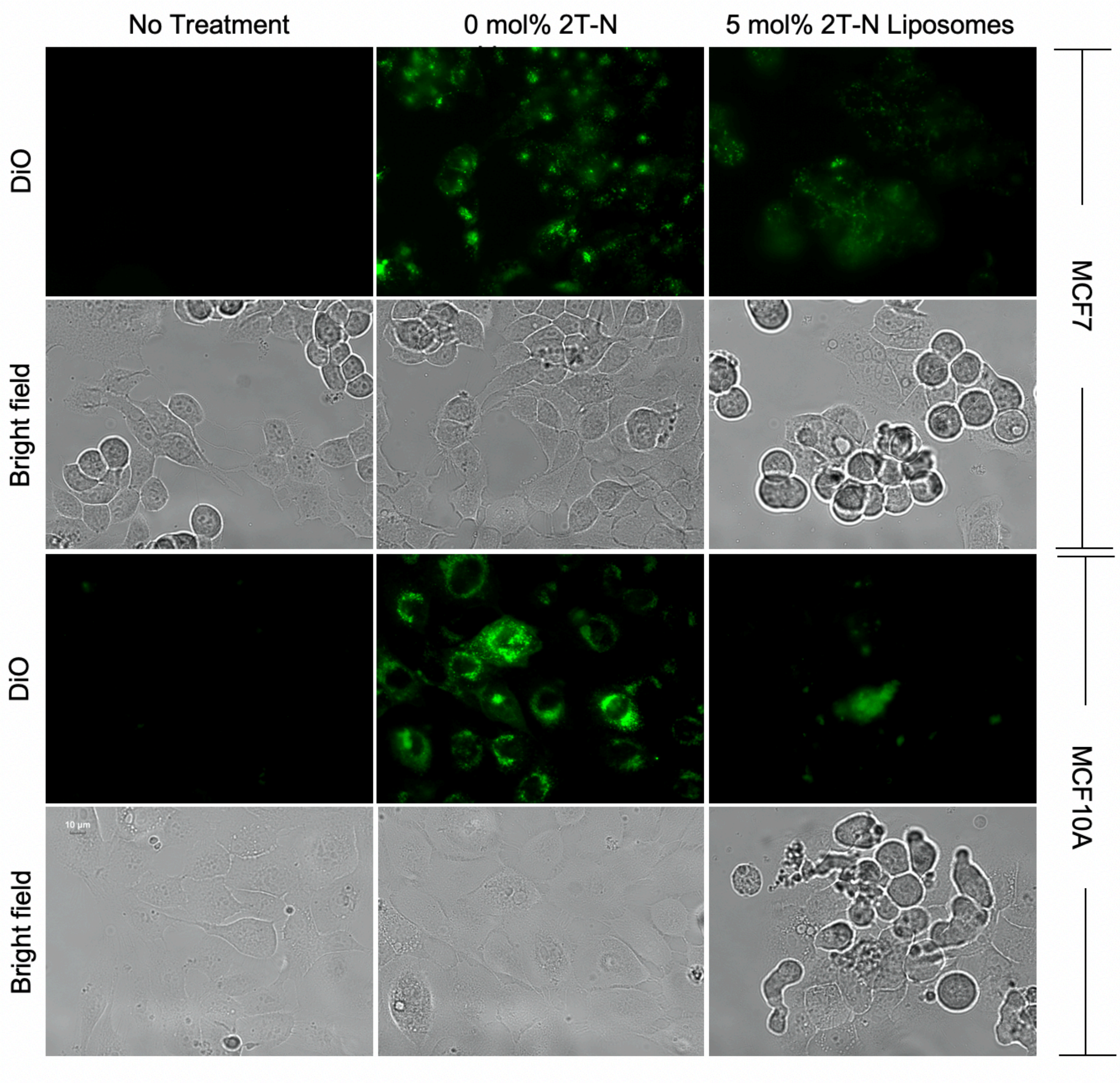

Figure S12: Liposome *in vitro* cellular uptake in MCF-7 and MCF10A cell lines post 24 hour incubation (2.1 μM of 2T-N).
